# Supplementary material for: Development of a Novel Competitive qRT-PCR Assay to Measure Relative Lentiviral Packaging Efficiency
Source: Mol Ther Methods Clin Dev. 2020 Sep 21;19:307–19. doi: 10.1016/j.omtm.2020.09.010 (PMC7581820; doi:10.1016/j.omtm.2020.09.010)
Supplement: Document S1. Figures S1–S6 [file mmc1.pdf]

OMTM, Volume *19*

Supplemental Information

Development of a Novel Competitive qRT-PCR Assay to Measure Relative Lentiviral Packaging Efficiency

Eirini Vamva, Andrew M.L. Lever, Conrad A. Vink, and Julia C. Kenyon

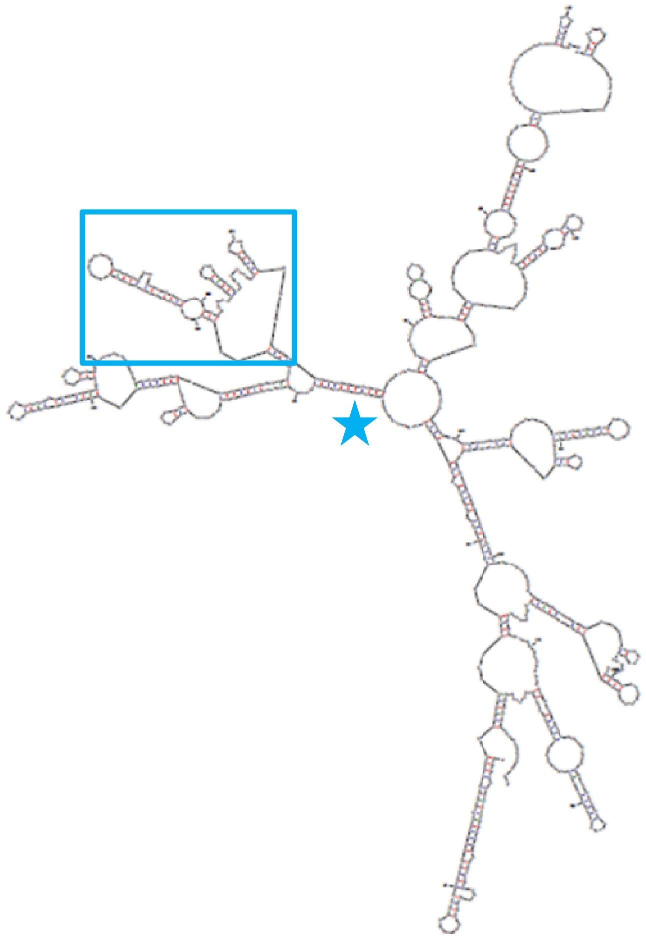

U5s (ΔG=-240.6 kJ/mol)

mFold predicted secondary structures of U5s mutant

Sup1

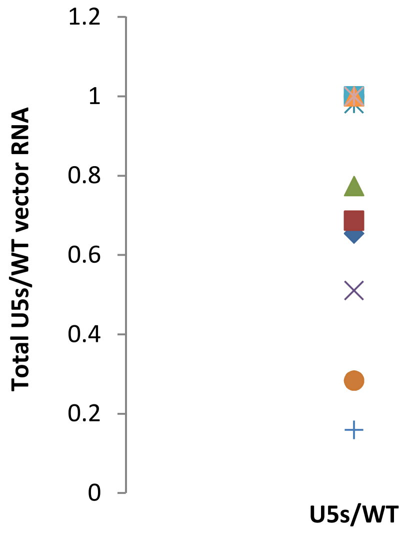

Alternative way of presenting U5s/WT packaging efficiency data from Northern blots in the form of individual data points.

Sup2

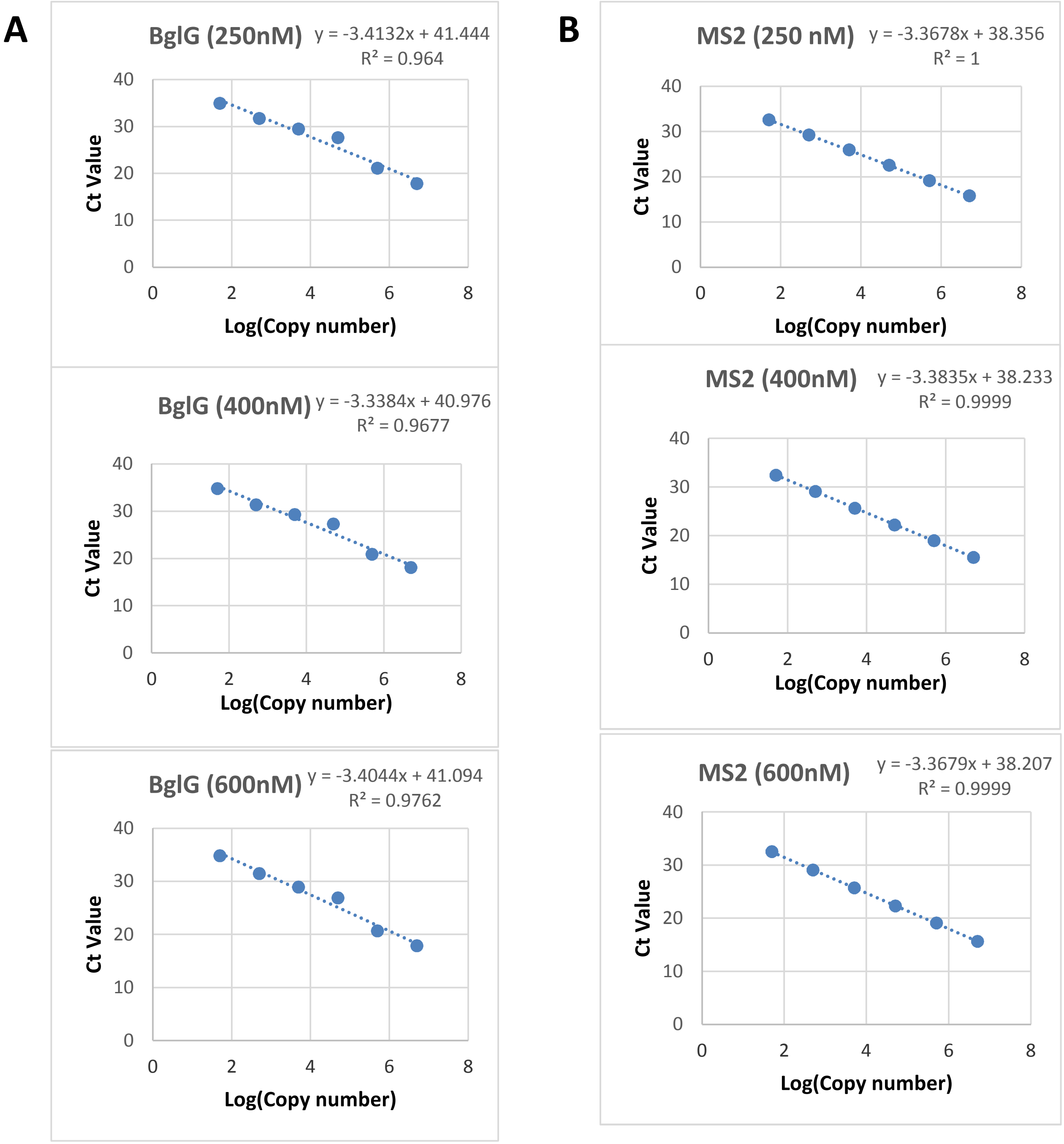

Optimization of [A] BglG and [B] MS2 probes' concentration

Sup3

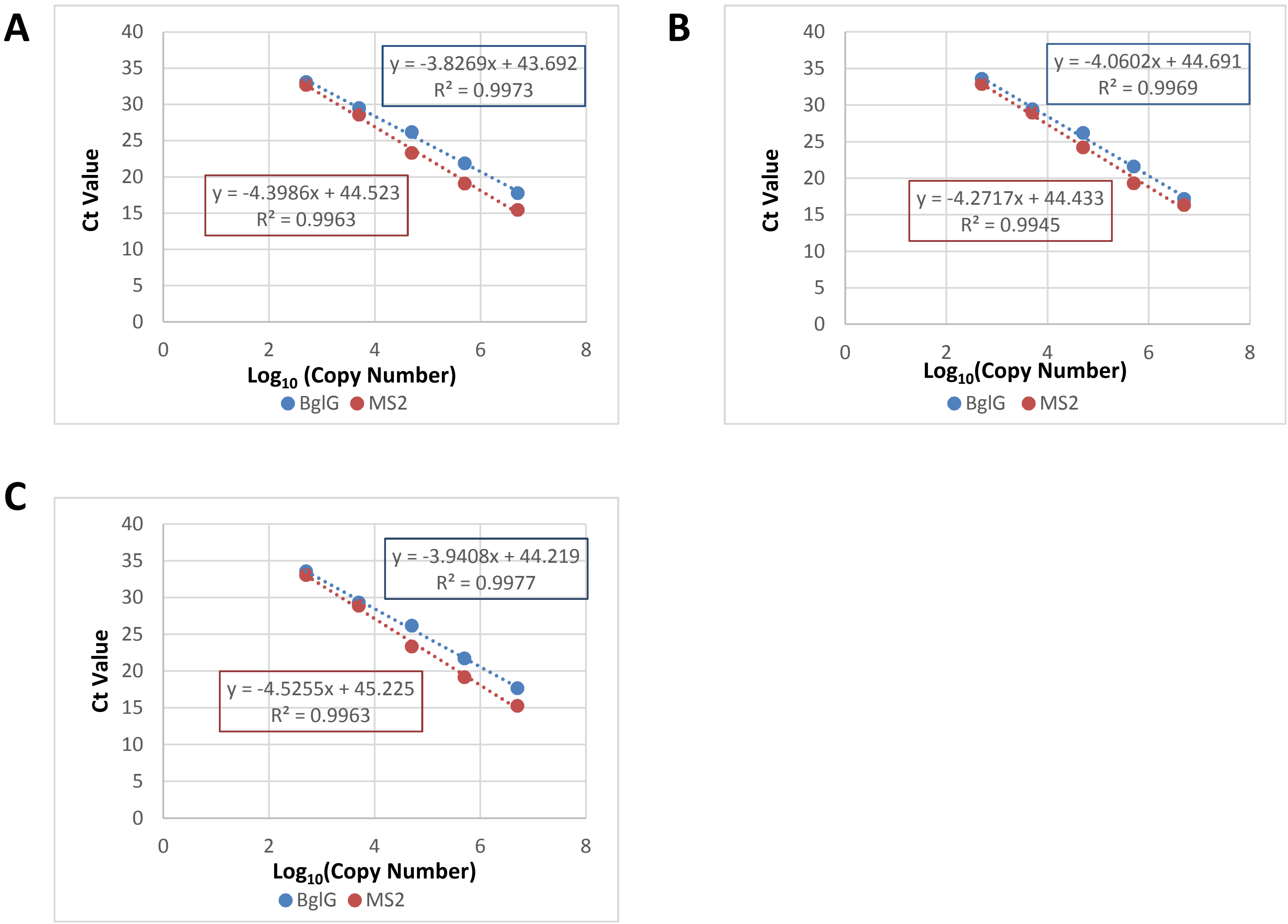

Multiplex reaction set up with 750 nM new AB BglG probe, 250 nM MS2 probe, 500 nM Forward primer, 500 nM Reverse primer and 5 µl Fast Advanced Buffer. [B] Multiplex reaction set up with 750 nM new AB BglG probe, 250 nM MS2 probe, 1000 nM Forward primer, 1000 nM Reverse primer and 5 µl Fast Advanced Buffer. [C] Multiplex reaction set up with 750 nM new AB BglG probe, 250 nM MS2 probe, 1500 nM Forward primer, 1500 nM Reverse primer and 5 µl Fast Advanced Buffer. Experiments shown are representative of 3 independent replicates, each using two technical repeats. The linearity of the 1000 nM MS2 assay (red) increased with increasing primer concentration whereas its efficiency decreased. On the contrary, the efficiency of the 1000nM BglG assay (blue) increased with increasing primer concentration, while the linearity of the standard curve decreased. The increase of primer concentration to 1500 nM positively affected linearity but negatively affected efficiency in both assays.

Sup4

Advantage / Technique	Length and integrity of target RNA	Sensitivity and accuracy	Throughput	Measurement of multiple RNA species	Level of internal control of the assay
Northern blot	+				
Ribonuclease protection assay (RPA)		+	+	+	
Standard RT-qPCR assay		++	++		
Competitive RT-qPCR assay		++	++	++	++

Comparison between techniques commonly used for measurement of RNA packaging efficiency and our new competitive RT-qPCR assay

Sup5

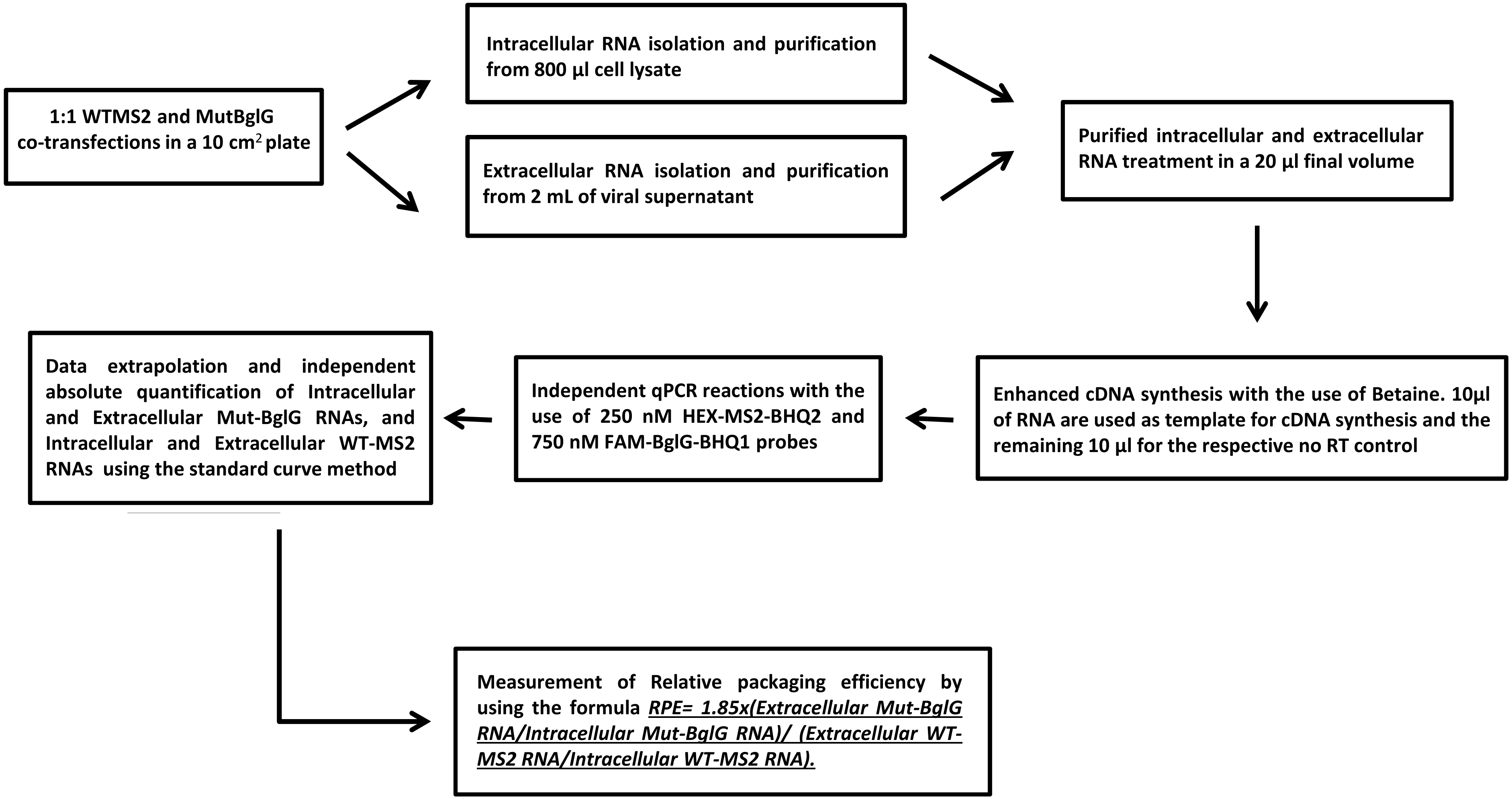

Flowchart of process required to measure RPE accurately

Sup6
