# Supplementary material for: Development of a Novel Competitive qRT-PCR Assay to Measure Relative Lentiviral Packaging Efficiency
Source: Mol Ther Methods Clin Dev. 2020 Sep 21;19:307–19. doi: 10.1016/j.omtm.2020.09.010 (PMC7581820; doi:10.1016/j.omtm.2020.09.010)
Supplement: Document S2. Article plus Supplemental Information [file mmc2.pdf]

Molecular Therapy
Methods & Clinical Development
Original Article

Development of a Novel Competitive qRT-PCR Assay to Measure Relative Lentiviral Packaging Efficiency

Eirini Vamva,[1,3] Andrew M.L. Lever,[1,2] Conrad A. Vink,[3] and Julia C. Kenyon[1,2,4]

[1]University of Cambridge Department of Medicine, Cambridge Biomedical Campus, Cambridge CB2 0QQ, UK; [2]Yong Loo Lin School of Medicine, 1E Kent Ridge Road, Singapore 119228, Singapore; [3]GlaxoSmithKline, Gunnels Wood Road, Stevenage SG1 2NY, UK; [4]Homerton College, Hills Road, Cambridge, CB2 8PH, UK

Third-generation HIV-1-derived lentiviral vectors are successfully used as therapeutic agents in various clinical applications. To further promote their use, we attempted to enhance vector infectivity by targeting the dimerization and packaging properties of the RNA transfer vector based on the premise that these two processes are tightly linked. We rationally designed mutant vectors to favor the dimeric conformation, potentially enhancing genome packaging. Initial assessments using standard assays generated outputs of variable reproducibility, sometimes with conflicting results. Therefore, we developed a novel competitive qRT-PCR assay in a co-transfection setting to measure the relative packaging efficiencies of wild-type and mutant transfer vectors. Here we report the effect of the dimerization-stabilizing mutations on infectious and physical titers of lentiviral vectors together with their packaging efficiency, measured using our novel assay. Enhancing dimerization did not automatically lead to better vector RNA packaging, suggesting that, for vector functionality, sufficient flexibility of the RNA to adopt different conformations is more important than the dimerization capacity. Our novel competitive qPCR assay enables a more stringent analysis of RNA packaging efficiency, allowing a much more precise understanding of the links between RNA structure, packaging, and infectious titers that will be invaluable for future vector development.

INTRODUCTION

Human immunodeficiency virus strain 1 (HIV-1) is the most well characterized of the lentiviruses, a family of viruses that reverse-transcribe and stably integrate their genetic information into the host genome. When used as lentiviral vectors (LVs), they can achieve gene delivery and sustained gene expression.[1] Vector RNA packaging predominantly depends on the 5′ RNA leader sequence of the virus, which is also the most conserved region of the HIV genome;[2] it contains *cis*-acting elements that regulate a variety of viral processes, including transcriptional activation, reverse transcription, splicing, and translation as well as genome dimerization and packaging[3,4] (Figure 1A). This 5′ leader RNA is present in its entirety in the HIV-1-based gene therapy vector system. To successfully assemble and release infectious viral particles, positive-sense RNA viruses like HIV-1 need to selectively recognize their genomic RNA (gRNA) from among a vast excess of similarly modified 5′-capped, 3′-polyadenylated spliced viral RNAs and cellular RNAs.[1] This specific packaging of the gRNA is mediated by the viral Gag protein, which recognizes particular RNA structures present in the 5′ gRNA leader.[5] Encapsidated gRNA is dimeric. Dimerization is necessary for efficient packaging of the gRNA and to produce infectious virions; however, the precise mechanism by which gRNA dimerization enhances packaging is the subject of ongoing debate.[6–11] A conformational switch between different monomeric structures in this gRNA leader region is thought to control the balance between translation of the gRNA to produce the Gag protein and capture of gRNA by Gag. The proposed monomeric RNA structures contain either an exposed gag start codon (AUG) for translation or an exposed dimerization initiation site (DIS), favoring dimerization and packaging.[12–14] The dimeric conformation is characterized by formation of several structural motifs, including the U5-AUG duplex (shown in Figure 1B). Mutations that strengthen U5-AUG base-pairing result in elevated levels of dimerization,[15,16] suggesting that this structural element promotes dimerization and, thus, packaging.

The HIV-1 genome has been modified extensively to increase its safety and efficiency as a vector for clinical applications. Despite it being an easy system for manipulation and production,[17] high vector doses and prolonged *ex vivo* culture conditions are required to achieve high transduction efficiencies of clinically relevant cellular targets, as exemplified in recent attempts to use LVs in clinical trials.[18–20] Currently, LV production is inefficient and expensive.[21]

We aimed to address this challenge by focusing on improving LV infectivity by creating mutants with enhanced packaging efficiency so that a higher percentage of vector particles would contain the correct transfer vector RNA. We created transfer vector mutants aiming to strengthen their ability to dimerize and package. Although the HIV-1 5′ leader has evolved to perform a vast number of functions

Received 2 April 2020; accepted 17 September 2020;
https://doi.org/10.1016/j.omtm.2020.09.010.

Correspondence: Andrew M.L. Lever, University of Cambridge Department of Medicine, Cambridge Biomedical Campus, Cambridge CB2 0QQ, UK.
E-mail: amll1@medschl.cam.ac.uk

This is an open access article under the CC BY license (http://creativecommons.org/licenses/by/4.0/).

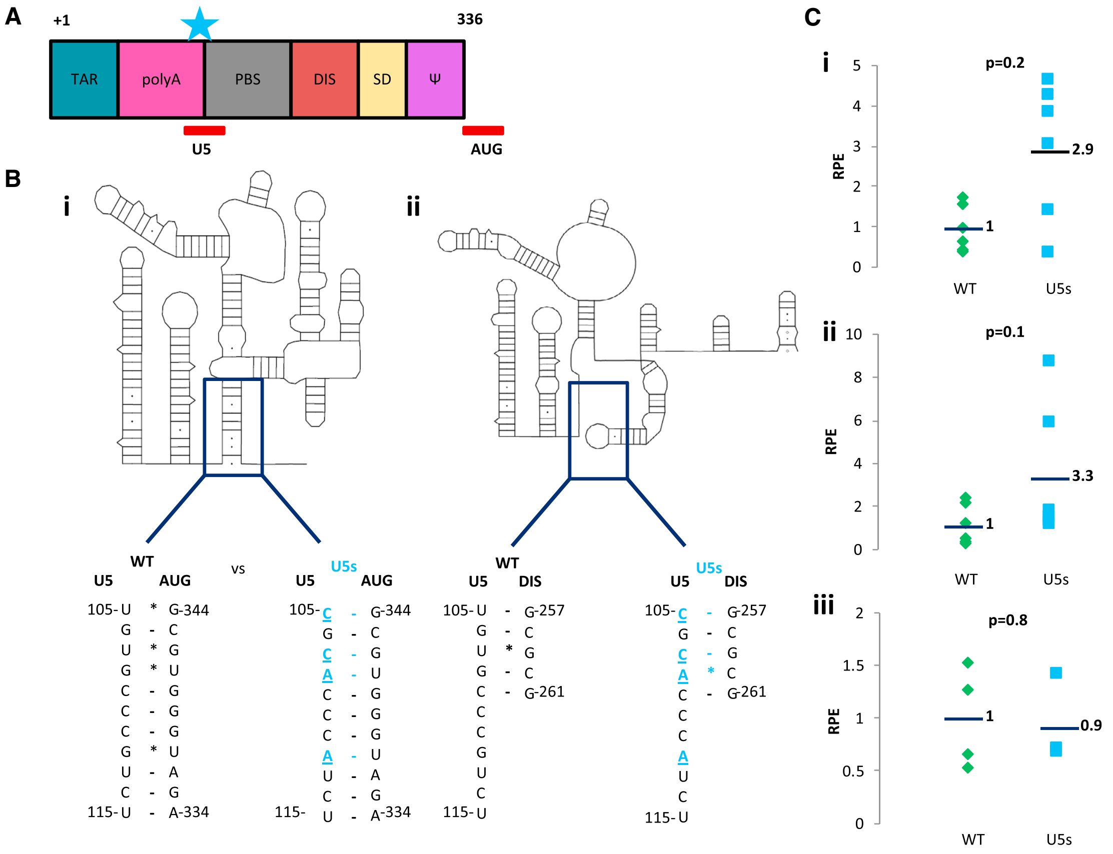

Figure 1. Design and Investigation of a U5-AUG-Stabilizing Mutant

(A) The *cis*-acting elements of the HIV-1 leader. The star indicates the region of the introduced mutations. (B) (i) Structural schematic of the dimerization-competent monomeric RNA structure of the HIV-1 leader. The U5s mutant was created by introducing mutations in the U5 region that strengthened the U5-AUG base pairing. 4 U-G non-canonical pairs were converted into two A-U and two C-G pairs, increasing the number of Watson-Crick hydrogen bonds by 6. (ii) Structural schematic of the dimerization-incompetent pseudoknot conformation of the monomeric RNA of the HIV-1 leader, which is characterized by the U5:DIS interaction. Point mutations are highlighted in light blue and underlined. (C) Packaging efficiencies of WT and U5s mutants. 293T cells were transfected with the WT or U5s transfer vector alongside the packaging plasmids. RNA was recovered and examined by qRT-PCR using a TaqMan probe specific for the WPRE region. Data are presented as packaging efficiency. Data show the packaging efficiencies of individual replicates of each of three separate experiments (i, ii, and iii). Horizontal lines show the average of each experiment.

in the context of HIV-1 infection, many of these are redundant in a vector system. In particular, because an individual HIV-1 gRNA molecule can be packaged without having itself translated first, the supply of Gag in *trans* means that the transfer vector 5′ leader does not have to retain the ability to promote translation.[22,23] We targeted the U5-AUG duplex because of its demonstrated importance for stabilization of the monomeric structure promoting dimerization[15,16] and its destabilization of the structure(s) promoting translation.[15,24]

With such an approach, it is critically important to be able to accurately determine the relative RNA packaging efficiencies of transfer vector mutants. Several methods exist to assess encapsidation of gRNA, including northern blot analysis, ribonuclease protection assay (RPA), and quantitative reverse transcriptase polymerase chain reaction (qRT-PCR) as well as the most recent sequencing technologies.[25] qRT-PCR is the most specific and sensitive of these methods[25] because it can accurately detect low-abundance transcripts in a complex transcriptome and is not reliant on full RNA integrity. However, for measurement of packaging efficiency, it suffers from technical biases because of the lack of established internal controls for normalization of vector RNA. Our initial results show that the data derived from such assays display poor reproducibility and, thus, cannot accurately determine relative packaging efficiencies. To overcome this limitation, we developed a novel qRT-PCR assay to measure the relative packaging efficiencies of transfer vectors in a competitive co-transfection environment. Here we report the effect of dimerization-enhancing mutations on infectious and physical titers of LVs as well as on their packaging efficiency, measured with this novel assay, which proved to be practical, accurate, and reproducible. Our data suggest that introduction of mutations that enhance RNA dimerization rather than packaging may have an adverse effect on the efficiency of genome encapsidation. This likely reflects the multifunctionality of the HIV-1 leader region and the possible effects of altering RNA flexibility on function. These findings regarding the

relationship between dimerization, packaging, and transduction efficiency provide further insights into this delicately balanced system and will help us to improve LV efficiency and enhance our capabilities to deploy these versatile vectors in clinical applications by improving their production.

RESULTS

Rational Design of a U5-AUG Mutant

Structures of the HIV-1 leader determined previously by in-gel selective 2'-hydroxyl acylation analyzed by primer extension (SHAPE)[24] were used to guide the design of U5-AUG helix-strengthening mutations aimed at enhancing dimerization of the RNA.[14,15,26,27] To strengthen the U5-AUG helix and enhance the proportion of RNA adopting this structure and, hence, being able to dimerize, it was necessary to consider the alternative structures the RNA has been shown to adopt. One conformation proposed to enhance translation and hamper packaging includes a pseudoknot between the U5 region and the DIS in the monomer.[16,24] Mutations were therefore designed to enhance the U5:AUG pairing without enhancing the stability of the U5:DIS pseudoknot (Figure 1B).

The overall structural effect of various base-pair changes in U5-AUG was assessed by minimal free energy structural prediction using the mFold[28] algorithm and the first 800 nucleotides of the HIV-1 HXB-2 RNA sequence.[29] Because this tool alone does not predict the monomeric pseudoknot structure even in wild-type (WT) RNA, the potential for each mutated sequence to form the pseudoknot was also assessed individually by comparing the pairing abilities of the U5 and DIS sequences (Figure 1B). The best-fit mutation for further study, U5s, is also shown in full in Figure S1 and was chosen based on the minimal free energy of its predicted dimeric structure, conservation of the global structure of the main packaging signal region (SL1-3), and its lack of ability to form a more stable U5:DIS interaction.

Use of Standard qRT-PCR to Measure RNA Packaging of the U5s Mutant Gives Highly Variable Results

WT and U5s transfer vectors were transfected independently into 293T cells with the plasmids encoding the packaging constructs. Two days after transfection, intracellular and virion RNAs were harvested and purified. Initially, a qPCR assay that amplifies the woodchuck posttranscriptional response element (WPRE),[30] a sequence inserted in the 3′ UTR of the LV backbone to enhance transgene expression,[31] was targeted to assess the effect of the U5s mutation on the packaging efficiency of the transfer vector. This assay was chosen over other qPCR assays in the literature, which mainly target sequences in the 5′ UTR of HIV-1,[32–36] to prevent interference between the 5′ UTR-located mutations affecting the assay's efficiency. Calculations were performed using the following equations:

Relative β-actin level for each sample = β-actin level/average β-actin level of all samples in the experiment; intracellular vector RNA (vRNA) level for each sample = vRNA level/relative β-actin level; packaging efficiency of each sample = extracellular vRNA level/intracellular vRNA level; relative packaging efficiency = packaging efficiency of mutant RNAs/packaging efficiency of WT RNAs.[37]

Three independent rounds of transfections of 4–6 samples were performed for the WT and U5s transfer vector mutants. The resulting data were highly variable, with the first round showing a statistically significant increase in the relative packaging efficiency of U5s compared with the WT ($p = 0.02$ by t test; Figure 1Ci), which was not corroborated in subsequent rounds (Figures 1Cii and 1Ciii). Using this protocol, at the virion RNA extraction stage, the RNA is present in such tiny amounts that its concentration cannot be determined, and it is precipitated with the aid of excess tRNA. This standard protocol is thus a multiple-step process that lacks an internal control necessary to assess extracellular vRNA recovery efficiency, potentially leading to the observed variability. This raised concerns regarding the assay's reproducibility and validity.

Relative Packaging Efficiency of the U5s Mutant Is Lower Than that of the WT When Measured by Northern Blot, Despite an Increase in Dimer Formation

To further investigate the properties of the U5s mutant, transfections were repeated as above, and RNA extracted from WT and U5s supernatants was analyzed by northern blot as an alternative method to visualize the amount of packaged RNA (Figure 2A; Figure S2) and calculate the propensity of their transfer vRNAs to dimerize *in virio* (Figures 2B and 2C). Densitometric analysis of northern blot RNA levels showed that U5s gRNA was packaged approximately two-thirds as well as WT vRNA (Figure 2A; $p = 0.009$ by t test), as analyzed by densitometric analyses of 7 replicates. Figure 2B shows a typical northern blot, with its densitometric analysis shown in Figure 2C. Increased dimerization was observed in each of 7 independent replicates (data not shown), averaging around 17% more dimer in U5s virions than WT virions ($p = 0.035$ by t test). The 690-nt-long *in-vitro*-synthesized RNA fragments of the transfer vectors under interrogation included all known *cis*-acting elements contained in the HIV 5′ leader and *gag* backbone of the third-generation HIV-1-derived LVs. Vector titration was then performed to study the relationship between packaging efficiency and transduction efficiency. Similar to the decreased packaging efficiency of U5s we observed in the northern blots, the infectious titer of the mutant transfer vector was decreased more than 1.5-fold compared with the WT ($p = 0.05$ by t test; Figure 2D). The discrepancy in results between the standard PCR-based packaging assay and the northern blot, combined with the transduction data, suggests that, despite the successful increase in dimerization upon stabilization of the U5:AUG duplex in U5s, its packaging and transduction efficiencies were impaired. The data also suggest that the standard packaging assay produces data that are too variable to reliably assess anything other than large differences in packaging efficiency.

The intracellular capsid-p24 expression level was quantitated by ELLA to detect any effect of the various 5′ UTR mutations on intracellular Gag expression and/or its ability to bud from the cell (Figure 2E). There were no observable or statistically significant

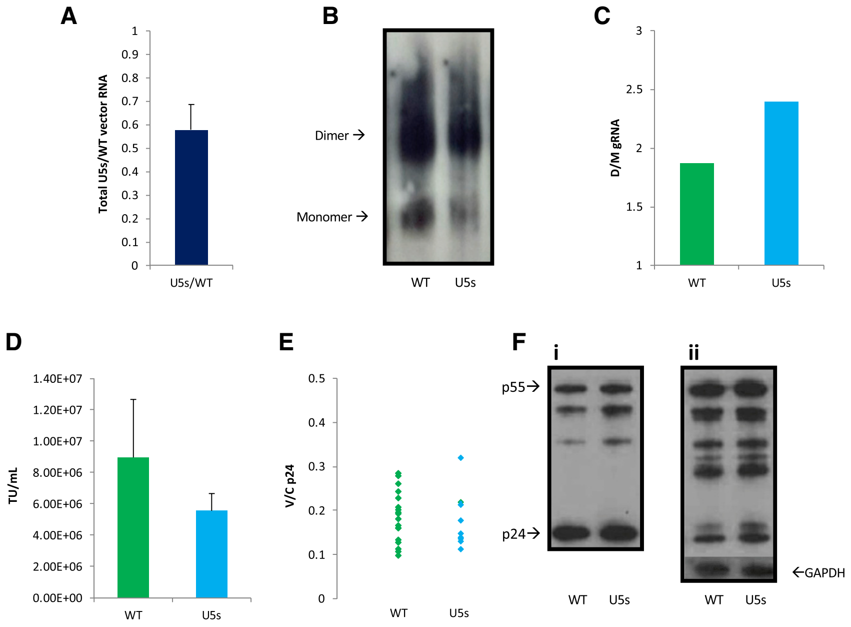

Figure 2. Further Characterization of WT and U5s Mutants

(A) Comparison of the total amount of encapsidated vRNA in the northern blots between WT and U5s transfer vectors by densitometry. Error bars show standard error. (B) Representative northern blot. 293T cells were transfected, and virions were purified from harvested supernatant by density gradient ultracentrifugation. RNA was extracted from equal volumes of WT and U5s vector supernatant, electrophoresed on a TBE gel, blotted, and visualized with a DIG-labeled probe. (C) Densitometric analysis of transfer vRNA dimerization in the northern blot shown in (B). (D) Comparison of the transduction efficiency of 293T cells with virions produced by WT or U5s viral vectors. Transduction units (TUs) were measured using flow cytometry. Data represent 7 repeats for the WT and 6 repeats for U5s from two independent rounds of transfections ($p = 0.05$). (E) Evaluation of the effect of 5′ UTR mutations on Gag budding by measuring the ratio of extracellular virion to intracellular Gag expression levels (V/C p24) with ELLA p24. (F) Pellets from WT and U5s supernatant and cellular lysates were examined by SDS-PAGE and anti-Gag immunoblots. GAPDH was used as a loading control for protein normalization across lysate samples.

differences in extracellular (vector [V]) to intracellular (cellular [C]) Gag quantities (p24 V/C) measured between the WT and the U5-AUG mutant ($p = 0.1$ by t test). In addition, Figure 2F suggests that Gag expression levels were equivalent in the WT and mutated constructs when assessed by immunoblot (no statistically significant differences were observed in total Gag levels or in the amount of each cleavage product between WT and U5s mutants following densitometric analysis of four independent experiments; data not shown). The unidentified bands in the intracellular samples are typical of the HIV Gag proteolytic products made by the vector system. Interestingly, the Gag p55/p24 antibody detected uncleaved p55 product in WT and U5s viral supernatants, which indicates the presence of immature particles and might reflect the presence of virus like particles (VLPs) lacking gRNA, highlighting the importance of improving the RNA packaging properties of the currently used transfer vectors. The terminal Gag processing step, p25 to p24 cleavage, is most sensitive to detect processing defects[38] but did not differ between the WT and U5s (Figure 2F). Overall, these results suggest that 5′ UTR mutations in the transfer vRNA do not affect Gag provided in *trans*; hence, any effect on the functionality of the mutant transfer vector was not caused by Gag expression, processing, or budding.

qPCR Assay Design and Optimization to Accurately Measure Relative Packaging Efficiency

Assessment of the relative packaging efficiencies (RPEs) of U5s and the WT transfer vector produced contradictory data from two different assays. The WPRE qPCR assay described above was characterized by large variability, possibly because of lengthy processing of samples and lack of an internal control for the extracellular RNA. Although retroviruses, including HIV-1, are known to recruit host cell RNAs into virions, the spectrum of encapsidated RNAs, the amount of specific RNAs packaged, and the mechanisms by which they are recruited remain largely unknown; hence the lack of an internal control for virion gRNA. Other available qPCR assays[32–36] would be expected to suffer from the same drawback. Northern blotting produced packaging efficiency data consistent with the transduction efficiency, but it is a much lower-throughput assay than qPCR, and its sensitivity is even more critically dependent on RNA quality and virus titer yield.[25] Hence, an alternative approach was needed to conclusively assess the packaging efficiency ratio for each construct. To achieve this, a competitive qPCR assay was designed by introducing unique sequences in the WT and mutant transfer vectors to allow simultaneous quantification of their vRNAs in a co-transfection environment. Such an environment controls for differences in cell viability as well as for recovery of the RNA in later steps of the protocol.

The MS2 bacteriophage sequence (5′-ACATGAGGATCACCCATGT-3′) and *E. coli* BglG sequence (5′-GGATTGTTACTGCATTCGCAGGCAAAACC-3′) were introduced separately into the WT and mutant U5s vectors (Figure 3A). The BglG and MS2 sequences form stem loops that, despite their complexity and potential effect on the respective qPCR assays, were chosen because they have been validated extensively in the literature as sequences that do not have a direct effect on the retrovirus life cycle.[27,39] A single copy of the MS2 or BglG sequence was introduced into a multiple cloning site (MCS) between the EGFP and WPRE elements. Cloning of these sequences individually into the WT and mutant constructs allowed independent quantification of their vRNA levels in a co-transfection context. The primer pair was the same for the BglG and MS2 constructs, whereas different probes were designed to target the unique MS2 and BglG sequences, allowing simultaneous quantitation of WT and mutant transfer vRNA levels in a one-well reaction.

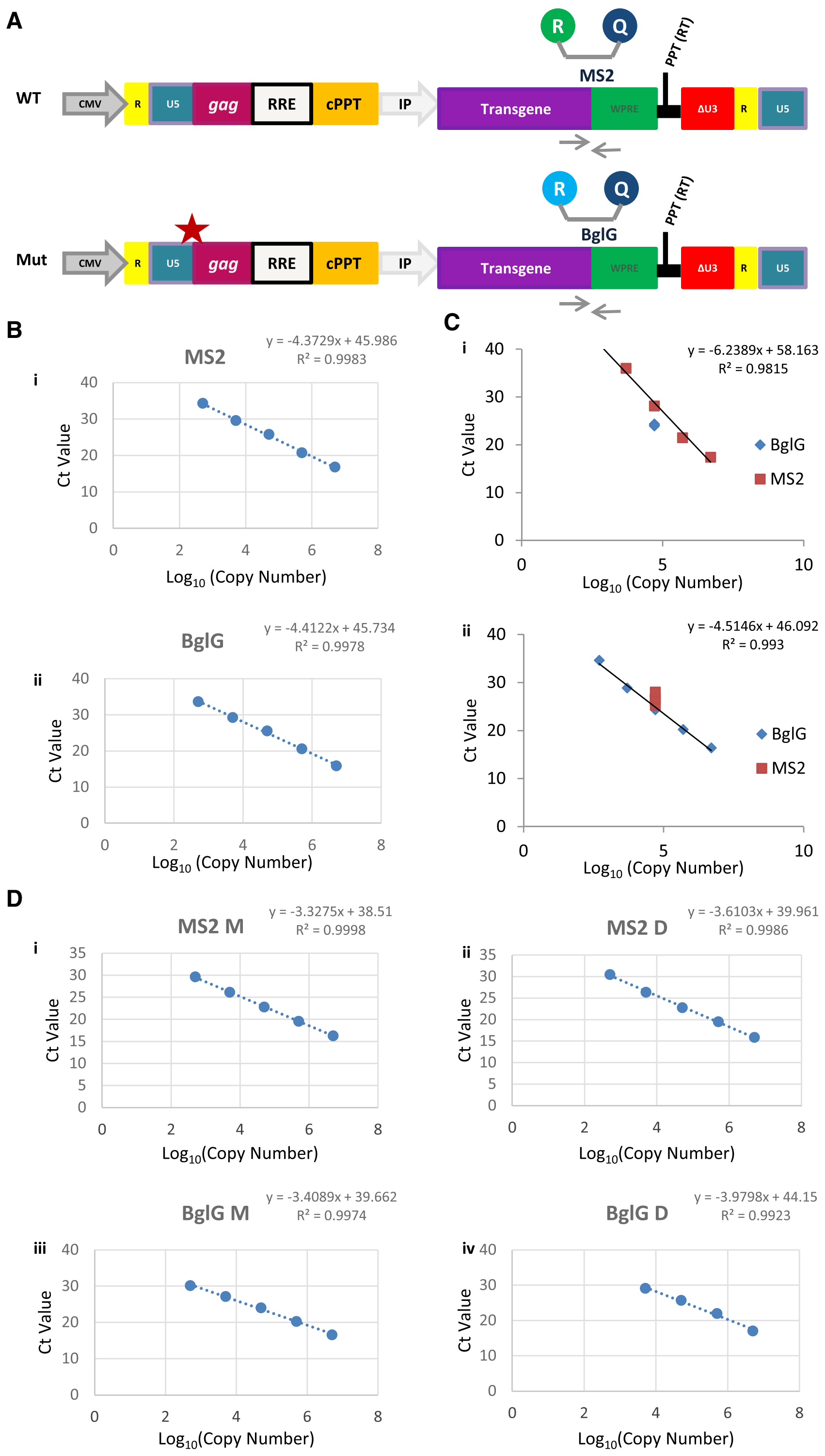

Figure 3. Design and Optimization of a Competitive qRT-PCR Assay

(A) Components of a typical third-generation lentiviral transfer vector. Shown is a schematic of the location of the introduced mutations (red star) and the unique sequences of MS2 and BglG stem loops as well as the binding sites of the common pair of primers. CMV, cytomegalovirus promoter; RRE, Rev-responsive element; cPPT, central polypurine tract; IP, internal promoter; WPRE, woodchuck posttranscriptional response element; R, reporter; Q, quencher. (B) Effect of the BglG and MS2 probe sequences on assay linearity and efficiency. The monoplex assay used 750 nM BglG probe (i) or 250 nM MS2 probe (ii), 500 nM forward primer and 500 nM reverse primer. 5×10^6 to 5×10^2 copies of WT-pCCL-EGFP-BglG plasmid and WT-pCCL-EGFP-MS2 plasmid were used as the respective standard curve templates. (C) Effect of constant and variable plasmid concentrations on MS2 and BglG assays to study any interference bias in a multiplex environment. Reactions used 750 nM new AB BglG probe, 250 nM MS2 probe, 1,000 nM forward primer, and 1,000 nM reverse primer and included (i) a constant of 5×10^4 copies WT-pCCL-EGFP BglG plasmid in the presence of a $\log_{10}$ WT-pCCL-EGFP-MS2 standard curve consisting of 5×10^6 to 5×10^2 copies or (ii) a constant of 5×10^4 copies WT-pCCL-EGFP MS2 plasmid in the presence of a $\log_{10}$ WT-pCCL-EGFP-BglG standard curve consisting of 5×10^6 to 5×10^2 copies. (D) Comparison of monoplex and duplex reactions for MS2 and BglG assays. The linearity and efficiency of the multiplex assay and the two respective monoplex assays were assessed in the same qRT-PCR plate. Assay linearity was assessed by R^2, whereas assay efficiency was calculated by standard curve slope. (i) Graph showing a five $\log_{10}$ standard curve of the MS2 assay in a monoplex reaction containing 250 nM MS2 probe, 500 nM forward primer, and 500 nM reverse primer. (ii) Graph showing a five $\log_{10}$ standard curve of the MS2 assay in a multiplex BglG-MS2 reaction containing 750 nM new AB BglG probe, 250 nM MS2 probe, 1,000 nM forward primer, and 1,000 nM reverse primer. (iii) Graph showing a five $\log_{10}$ standard curve of the BglG assay in a monoplex reaction containing 750 nM new AB BglG probe, 500 nM forward primer, and 500 nM reverse primer. (iv) Graph showing a five $\log_{10}$ standard curve of the BglG assay in a BglG-MS2 multiplex reaction containing 750 nM new AB BglG probe, 250 nM MS2 probe, 1,000 nM forward primer, and 1,000 nM reverse primer. The experiments shown in (i) and (iii) were performed 13 times; those shown in (ii) and (iv) were performed twice. All results were similar.

MS2 and BglG targeting probes were assessed according to the MIQE (minimum information for publication of qRT-PCR experiments) guidelines.[40] Calibration of primer concentration was achieved by maintaining the MS2 probe concentration constant and varying the primer concentration, resulting in identification of 500 nM of each primer as the optimal primer concentration that achieved a low cycle threshold (Ct) value and lack of product formation in the respective negative control wells (data not shown). Using this primer set concentration, probe concentrations were optimized. The WT-pCCL-EGFP-MS2 and WT-pCCL-

EGFP-BglG plasmids were used as standard curve templates in independent monoplex reactions to interrogate the efficiency and linearity of the assays. Using our initial design of MS2 probe, all probe concentrations had an R^2 value of more than 0.99. The PCR efficiency was approximately 98% for all MS2 reactions and 97%–100% for the BglG reactions (Figure S3). BglG probe sequence optimization was required because, as shown in Figure S3, for our initial BglG probe design, all of the probe concentrations showed a variability between 5×10^5 and 5×10^3 copy numbers without achieving an acceptable level of linearity for BglG; all probe concentrations also generated an R^2 that was lower than 0.99. To improve this, a new 6-carboxyfluorescein (FAM)-labeled BglG probe was designed (Applied Biosystems[41] Custom TaqMan MGB Probe Design Service, 5′-TCGATCGGGATTGTTACTG-3′) and was used in the assay at a concentration of 750 nM, as directed by the manufacturer. Keeping the primer concentration at 500 nM, as established previously, independent monoplex reactions for MS2 (250 nM) and the new BglG (750 nM) were performed using the MS2 and BglG plasmids, respectively, for templates and were assessed for linearity and efficiency (Figure 3B), showing acceptable characteristics.

The BglG and MS2 assays were initially optimized in monoplex reactions, where RNAs from cotransfection experiments were processed as one sample until the last stage of the qPCR experiment, and then MS2 and BglG signals were detected in two separate wells of the qPCR plate. Because it should be possible and faster to detect MS2 and BglG signals in the same well of the qPCR assay, we attempted to optimize the assay in this multiplex form. We first analyzed whether the primer concentration needed to be reoptimized in the multiplex assay. The effect of the 500 nM primer concentration, which is the optimal primer concentration established in the monoplex assays described previously, was compared with 1,000 nM and 1,500 nM primer concentrations, but as presented in Figure S4, the resulting data showed that no primer concentration in the multiplex assay amplified in a manner that was as linear as in the monoplex assay. Additionally, the comparable efficiencies of MS2 and BglG observed in Figure 3B were affected negatively, as noted by the increased difference in the amplification factors of these two assays in Figure S3. We concluded that amplification could have been affected by primer and probe interactions, competition for reagents between the two amplicons of slightly different sizes within the same well reaction, as well as the relative target sequence expression levels, resulting in more stochastic detection in the multiplex environment compared with the monoplex reactions.

A new set of experiments was designed, maintaining the plasmid concentration of WT-pCCL-EGFP-BglG at a constant of 5×10^4 copies/well in the presence of a $\log_{10}$ WT-pCCL-EGFP-MS2 standard curve and vice versa. This experiment was performed to reflect the wide differences in template availability that might occur when comparing the packaging efficiencies of two vectors with very different packaging abilities. This time, the efficiency and linearity of each assay were investigated by the Ct consistency of the plasmid whose DNA mass was kept constant. The Ct difference of a 10× sample dilution was expected to be around 3.2. As shown in Figure 3C, the slope of the MS2 standard curve in the presence of a constant amount of BglG plasmid was 6.2 (with variations between individual 10× dilutions of between 4.1 and 7.9 Ct values), which was of poor efficiency. Respectively, the slope of the BglG standard curve in the presence of a constant amount of MS2 plasmid was 4.5 (with the difference between individual 10x dilution values varying between 3.8 and 5.7 Ct values). As expected, the Ct value of the constant amount of BglG plasmid was maintained at 24, between 6.7 and 2.7 of log10 (copy number) of MS2 DNA. The Ct value of the MS2 plasmid kept at a constant level was measured between 25.3 and 26.6 between 6.7 and 3.7 of log10 (copy number) BglG copies but at 28 in the presence of 2.7 log10 (copy number) of BglG copies.

Given the data presented in Figure 3C, reevaluation of the use of an independent monoplex assay versus a multiplex assay was performed by directly comparing the linearity and efficiency of the multiplex assay and the two respective monoplex assays in the same qRT-PCR plate. The monoplex and multiplex assays use MS2 and BglG sequence-containing genetic material derived from the same co-transfection plate. All processing steps are identical until the final stage of the qRT-PCR assay, where the sample is split in the final step of the process at the level of qPCR. A monoplex assay thus maintains the competitive nature of the experimental environment and still excludes the potential variability introduced by the processing steps of the standard WPRE assay. Figure 3D shows that the MS2 monoplex qPCR efficiency was calculated to be 99.77% with an amplification factor of 2 and an R^2 of 0.999, whereas the equivalent MS2 multiplex qPCR efficiency dropped to 89.23% with an amplification factor of 1.89 and an R^2 of 0.998. Similarly, the BglG monoplex qPCR efficiency was 96.49% with an amplification factor of 1.96 and an R^2 of 0.997, whereas the equivalent BglG multiplex qPCR efficiency was decreased to 78.35% with an amplification factor of 1.78 and an R^2 of 0.992. Collectively, the data confirm that the equivalent monoplex assays were more efficient than the multiplex reaction. The multiplex assay would be preferable in terms of time and cost efficiency but was not found to be as accurate as the monoplex assays. Therefore, independent use of the optimized monoplex MS2 and BglG assays was chosen for further study of the U5:AUG mutants.

Calibration of the Novel Packaging Assay in a Cellular Context

The experimental process followed to measure RPE with our novel competitive qPCR assay is depicted in Figure 4A. This method overcomes the necessity to normalize samples to a housekeeping gene because the genetic material from the WT and mutant vectors comes from the same transfection environment. An MS2 and a BglG version of the WT and U5s transfer vectors were constructed. We then sought to investigate whether the assay was equally efficient at detecting BglG- and MS2-containing cDNA produced from extracted vRNA. This was important to ensure unbiased and accurate measurement of WT and mutant vRNA packaging regardless of the incorporated heterologous sequence. To study this, HEK293T cells were co-transfected with equal amounts of WTBglG and WTMS2 transfer vector plasmids alongside the packaging constructs. Viral gRNA was extracted from 2 mL of viral supernatant. All reverse transcription

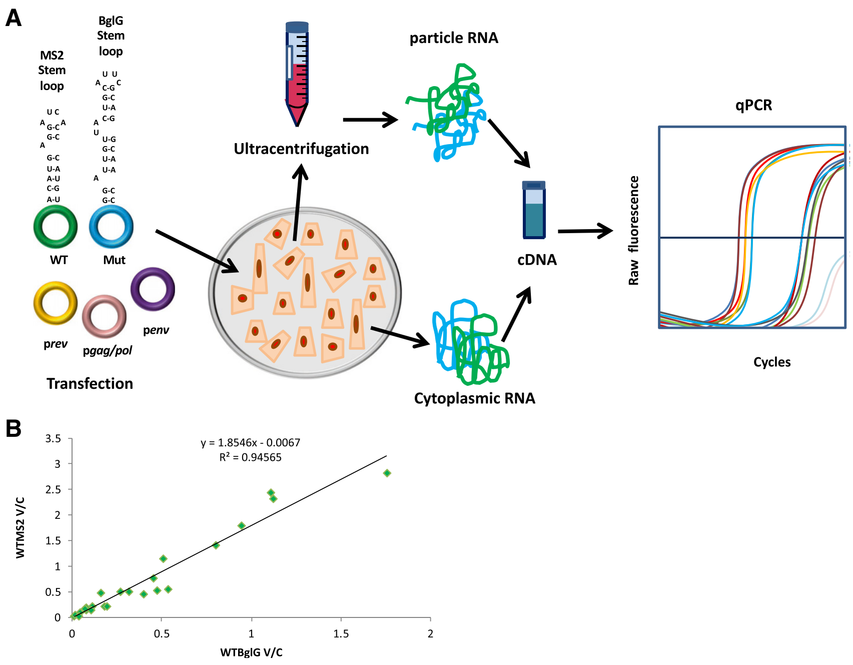

Figure 4. Assessment of Packaging Efficiency Using the Competitive qRT-PCR Assay

(A) Schematic showing the experimental workflow followed to assess packaging efficiency with the novel competitive qRT-PCR assay. 293T cells were transfected with equal amounts of WT and mutant transfer vector plasmids alongside the packaging constructs. RNA was extracted from viral supernatant and cell lysates, DNase treated, and used as template for qRT-PCR. RPE was defined as the ratio of extracellular to intracellular WT gRNA divided by the ratio of extracellular to intracellular mutant gRNA derived from the same co-transfection plate. (B) Linear distribution of the WT-pCCL-EGFP-MS2 and WT-pCCL-EGFP-BglG extracellular to intracellular ratio values (V/C). The average RPE reflecting the MS2/BglG assay sensitivity was calculated to be 1.85.

reactions contained 0.5 M betaine to enhance RT efficiency through regions of strong RNA secondary structure.[42] The extracellular-to-intracellular gRNA ratios of WTMS2 and WTBglG were plotted against each other to study the distribution of their values, which, as shown in Figure 4B, fall in a linear distribution. The average WTMS2/WTBglG efficiency from two independent experiments was calculated to be 1.85 from 33 independent transfection replicates, with a standard deviation of 0.34. Thus, in all further experiments, we divided the MS2 result by 1.85 (whether the MS2 labels the WT or mutant) before then taking the ratio between the WT and mutant. This takes into account the inherently lower BglG assay efficiency. Users should apply this adjustment before calculating the RPE. The WT or mutant can be labeled with the MS2 sequence.

Validation of Assay Sensitivity to Measure Different Levels of Packaging

Co-transfections of the U5sBglG/WTMS2 and U5sMS2/WTBglG combinations were performed next to verify that the experimental protocol and analysis were optimally calibrated and able to produce data with minimal standard deviation. As shown in Figure 5, the average U5sBglG/WTMS2 RPE from 14 individual samples was measured to be 0.52. This was statistically significantly different from WTBglG/WTMS2 data, which averaged 1 with a standard deviation of 0.34 ($p < 0.0001$ by t test). If the two assays are equally efficient, then the expected U5sMS2/WTBglG RPE should give similar results. Figure 5 shows that the average U5sMS2/WTBglG RPE from 13 independent co-transfection repeats was indeed measured to be significantly lower than that of the WT at an RPE of 0.4 ($p < 0.0001$ by t test compared with WTMS2/WTBglG as above). These results validated the accuracy and robustness of this novel qPCR assay regardless of whether the WT or mutant vector contained the MS2 or BglG sequence (these results for U5sBglG/WTMS2 and U5sMS2/WTBglG were not statistically significantly different from one another; $p = 0.13$ by t test). Across four such experiments (2 of U5sBglG/WTMS2 and 2 of U5sMS2/WTBglG), the average RPE of U5s was 0.49 with a SD of 0.19. A power analysis suggests that users should perform at least 8 replicates of WT/WT and mutant/WT to reliably detect the difference in means between the WT and a mutant that packages around half as well; fewer replicates are needed to detect larger differences and more replicates for smaller differences.

We then proceeded to determine the assay's limit of detection (LOD) by calculating the RPE of a known packaging deficient mutant. ΔP1 is an HIV-1 mutant with a 19-bp deletion in the Ψ region whose packaging efficiency has been measured previously by different methodologies to be less than 2% of that of the WT virus.[43,44] The ΔP1-pCCL-EGFP transfer vector was created and modified to contain the BglG insert. The ΔP1-pCCL-EGFP-BglG plasmid was used for co-transfections with WT-pCCL-EGFP-MS2. From the 13 independent transfections, 6 replicates were excluded because their Ct values were very high (up to 37) and too close to background control levels to be analyzed, likely because of the established low packaging efficiency of this mutant. The remaining 7 were taken into consideration for further analysis. The average ΔP1 pCCL-EGFP-BglG/WT pCCL-EGFP-MS2 RPE of the replicates containing sufficient RNA to analyze was calculated to be 0.017 ($p = 0.003$), suggesting that, in comparison with the WT, a maximum of 1.7% of the ΔP1 gRNA was packaged successfully (Figure 5). This result confirms the suitability of the assay to accurately measure the RPE of mutants with a packaging efficiency as low as 1.7% of the WT.

Use of the Assay to Assess an Alternative U5-AUG Mutant

An alternative U5-AUG duplex-stabilizing mutant was designed previously by Lu et al.[16] and shown to favor dimerization *in vitro*. To

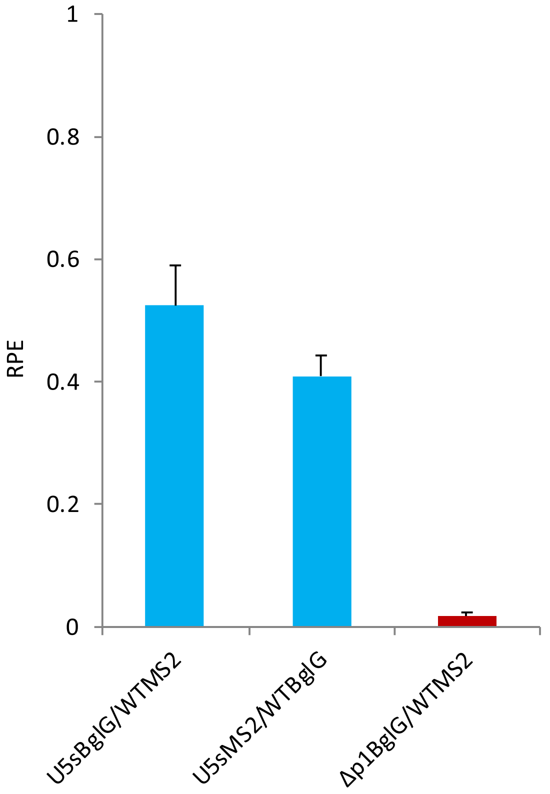

Figure 5. Depiction of the Average U5sBglG/WTMS2, U5sMS2/WTBglG, and ΔP1BglG/WTMS2 RPE Values

293T cells were transfected with equal amounts of WT-pCCL-EGFP-MS2 and U5s-pCCL-EGFP-BglG transfer vector plasmids, WT-pCCL-EGFP-BglG and U5s-pCCL-EGFP-MS2 transfer vector plasmids, and WT-pCCL-EGFP-MS2 and ΔP1-pccL-EGFP-BglG transfer vector plasmids alongside the packaging constructs. RNA extraction and purification was achieved as in Figure 1C, and RPE measurements with qRT-PCR were made following the workflow presented in Figure 4A. The assay's LOD was defined by ΔP1BglG/ WTMS2 RPE. Error bars show standard error.

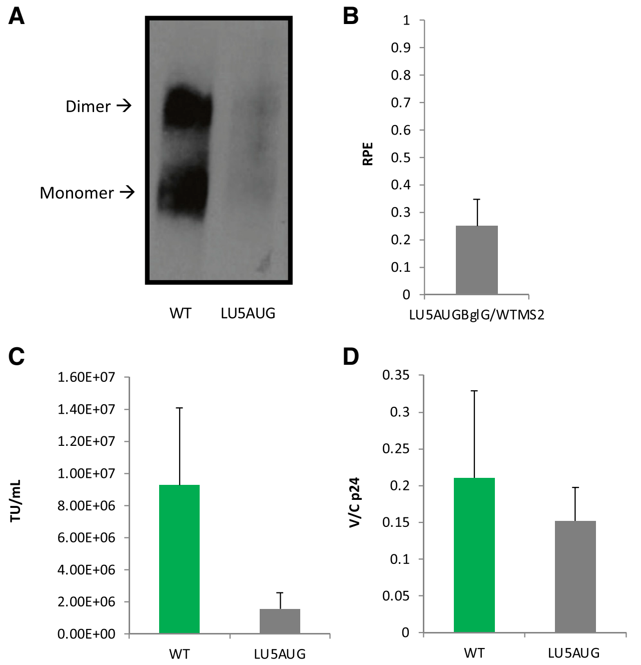

Figure 6. Characterization of the LU5AUG Transfer Vector Mutant

(A) Visualization of WT and LU5AUG vRNA by northern blot, performed as in Figure 2. (B) Measurement of WT and LU5AUG transfer vector average RPE from 8 independent co-transfection repeats, following the workflow presented in Figure 4A. Error bars show standard error (p = 0.0005). (C) 293T cells were transduced with varying dilutions of pseudotyped virions made using WT-pccL-EGFP or LU5AUG-pccL-EGFP transfer vectors. Infectious titers were calculated by flow cytometry of EGFP-expressing cells, using the dilution in which samples contained 4%–25% EGFP+ cells (p = 0.0002). (D) ELLA p24, performed on purified viral supernatants and cellular lysates. The V/C p24 ratio for WT and LU5AUG transfer vectors was measured to study any effects of 5′ UTR mutations on Gag budding. Error bars show standard deviation (p = 0.9).

determine whether the U5s mutant is unique in its ability to enhance dimerization but compromise packaging, the LU5AUG mutations were introduced into our pCCL-EGFP transfer vector construct to test its behavior in an *in virio* context.

RNA extracted from separately transfected cultures of WT and LU5AUG vector supernatants was examined by northern blot to compare the propensity of their gRNA to dimerize *in virio* with the *in vitro* dimerization efficiency published data. However, as shown in Figure 6A, measurement of the dimerization efficiency of LU5AUG could not be evaluated accurately because of poor yields of the LU5AUG vector. As shown in Figure 6B, use of our novel qPCR assay revealed a 5-fold reduction in the RPE of the LU5AUG mutant (p = 0.0005 by t test compared with WTMS2/WTBglG), again reflecting a negative effect of the dimerization-enhancing mutations on packaging in the context of a vector system. This relatively small decrease was sufficient to prevent LU5AUG vRNA visualization in virions, highlighting the limitations of the northern blot technique and emphasizing the importance of establishing a sensitive assay to study genome packaging. Transduction efficiencies of virions made from WT and LU5AUG constructs showed a 6-fold decrease in infectious titers compared with the WT (p = 0.0002 by t test; Figure 6C). Thus, similar to the U5s data, the LU5AUG mutant shows that there is not a simple and direct link between dimerization and packaging, as hypothesized originally, and such a result emphasizes the multifunctionality of this structural element of the HIV-1 leader, whose structural flexibility might be required for successful virus replication. Last, ELLA p24 was performed to measure intracellular and extracellular capsid-p24 expression levels, but the p24 V/C ratio measured showed no difference between the WT and LU5AUG (p = 0.9 by t test), confirming that the 5′ UTR mutations do not affect in *trans* Gag expression (Figure 6D).

DISCUSSION

The WT HIV-1 RNA sequence has evolved to be able to present multiple critical replication signals in the context of a complex leader

structure.[45] The HIV-1 gRNA 5′ region can adopt different structures depending on whether it is monomeric (assumed to be optimal for translation) or dimeric (required for efficient packaging). Rationally designed transfer vector mutants that favored the dimeric conformation and might enhance packaging were created by stabilizing the U5-AUG duplex located in the 5′ UTR. A variety of assays were performed to assess the effects of the introduced mutations on different parts of the vector life cycle. However, no available assays were sensitive and robust enough to measure the RPE of our U5s mutant compared with that of the WT. To address this, we designed a novel competitive qPCR assay to measure the effect of mutations on the packaging efficiency of mutant transfer vectors.

The U5s mutant, designed to have strengthened U5-AUG base-pairing, showed slightly lower infectious titers than the WT, whereas the LU5AUG mutant, designed with the same rationale but containing a larger number of point mutations, saw a bigger decrease in infectious titer. Western blotting showed no change in the Gag processing pattern of mutants compared with the WT, suggesting that introduction of 5′ UTR mutations does not affect the processing of Gag provided in *trans*, whereas measurement of the intracellular and extracellular p24 levels with ELLA p24 showed that the 5′ UTR mutations did not affect the amount of viral budding. To assess the effect of mutations on RNA dimerization efficiency, northern blotting was performed to visualize and measure the total amounts of encapsidated vRNA and its propensity to dimerize. This was initially chosen as the most appropriate technique because of its advantage of measuring the quantity of total encapsidated vRNA levels and the stability of its dimeric form. It revealed increased RNA dimerization for U5s but virtually no LU5AUG vRNA, presumably because of the lower packaging efficiency of this mutant.

Our competitive qRT-PCR approach offered a direct comparison between the WT and mutant transfer vectors because it allowed measurement of the RPE in a co-transfection environment, reducing the possibility of technical biases or sample processing errors influencing the result.

This novel competitive qRT-PCR assay was sensitive and accurate, able to detect an RPE of less than 2% of WT RNA as exemplified by the ΔP1 mutant, and detected smaller differences in encapsidation of mutants, like U5s, whose packaging efficiency was more comparable with that of the WT. The assay enabled our investigation of whether two different dimerization-enhancing mutations promote packaging, something that was not possible to do accurately using previously available techniques. Our results showed that enhancing dimerization does not automatically lead to better packaging of vRNA.

The propensity of both mutated vRNAs to dimerize had indeed increased as hypothesized. This result confirms the importance of the U5-AUG duplex in stabilizing the dimeric RNA conformation. The surprising deleterious effect on packaging may be due to the introduced mutations affecting multiple vector processes. This result reflects the multifunctionality of the 5′ leader sequence and the disruptive effects of interfering with the flexibility of it by stabilizing the U5-AUG duplex, suggesting that RNA structural flexibility may be more important than RNA dimerization propensity for efficient packaging. Measurement of transduction efficiencies revealed a negative effect of the rationally designed mutations on the infectivity of the pseudotyped vector that was commensurate with their reduced packaging phenotype. A different approach for future research would be to perform these infectivity experiments under conditions where availability of dimerized gRNA would be limiting for packaging. Use of dimerization-promoting mutants under limiting conditions might enhance their biological effects on packaging and improve our understanding of the mutant phenotypes. An improved understanding of the basic biology of the dimerization and packaging processes and the RNA structures involved is necessary for rational design of more efficient vectors for the demanding future of gene therapy, especially for their use in *in vivo* applications. Our novel competitive qPCR assay was found to be the most efficient and robust assay for accurate measurement of the RPE of transfer vectors (Figure S5) with the potential to also be used to measure transduction efficiencies in a mixture of viral vectors produced in a competitive co-transfection environment. Figure S6 includes a flowchart that illustrates how future users can best implement the qPCR assay in an optimal way and extrapolate values from raw data in the most reproducible manner. Such an assay adds an important asset to the current portfolio of techniques to investigate different parts of the LV life cycle and will help us to understand the biology of transfer vectors and ways to optimize them for clinical gene therapy.

MATERIALS AND METHODS

Lentivirus Production by Transient Transfection

293T cells were maintained in Dulbecco's modified Eagle's medium supplemented with 10% fetal bovine serum (Gibco) at 37°C. For each transfection 5×10^6 cells were seeded in 10 mL medium in a 10-cm^2 dish. The medium was changed at 18 h, and cells were transfected 20–21 h after seeding. For each transfection plate, 500 μL of 250 mM $CaCl_2$ containing a total of 25 μg DNA in a 1:1:1:3 molar ratio of pSYNGP:pRev:pVSVg:pCCL-EGFP transfer vector was added dropwise to 500 μL of 2× HEPES-buffered solution (pH 7; Alpha Aesar) while bubbling air through it and incubated at room temperature for 30 min. The solution was mixed and immediately added dropwise across the plate, followed by swirling to ensure even distribution. The medium was replaced with a 5-mL volume 16 h after transfection. Supernatant was harvested 40 h after transfection, filtered through a 0.2-μm filter, aliquoted into cryovials, and stored at −80°C. Transfection efficiency was assessed by flow cytometry detecting EGFP-positive cells. The minimum transfection level for continuation of experiments was 95%.

Vector Titration with Flow Cytometry

293T cells were seeded at 3×10^5 per well in a 6-well plate at a final volume of 2 mL. Viral supernatants were thawed and used to infect cells 18 h post-seeding at a series of dilutions in a final volume of 500 μL containing 8 μg/mL Polybrene. LVs were titrated 48 h after

transduction by flow cytometry assessment of EGFP-positive cells. Titers were calculated using transduced wells in which the proportion of EGFP-positive cells was between 4% and 25%. The transduction efficiency of each LV was calculated by multiplying the number of seeded cells by the percentage of EGFP-positive cells and by the vector dilution factor. This value was then divided by the volume of LV used for HEK293T cell transduction. Transduction units were defined as the number of functional viral particles in the supernatant that were capable of transducing a cell and successfully expressing the transgene.

Constructs and Primers Used

The Agilent QuikChange Primer Design Program[46] was used to create primer sequences for site-directed mutagenesis (SDM). Primers were as follows:

U5sFw, 5′-GCCTTGAGTGCTTCAAGTAGTGCGCACCCATCTGTTGTGTGACTCTGGTAAC-3′

U5sRv, 5′-GTTACCAGAGTCACACAACAGATGGGTGCGCACTACTTGAAGCACTCAAGGC-3′

LU5AUGFw1, 5′-CTAGCGGAGGCTAGAAGGAGAGAGACGGGCACAAGAGCGTCAGTAT-3′

LU5AUGRv1, 5′-ATACTGACGCTCTTGRGCCCGTCTCTCTCCTTCTAGCCTCCGCTAG-3′

LU5AUGFw2, 5′-GCTAGAAGGAGAGAGACGGGCACAAGAAAAAAGGTATTAAGCGGGGGAGAATTAGATCGC-3′

LU5AUGRv2 5′-GCGATCTAATTGTCCCCCGCTTAATACCTTTTTTCTTGTGCCCGTCTCTCTCCTTCTAGC-3′

ΔP1DelFw, 5′-CGGCGACTGGTGAGTACGCCGGCTAGAAGGAGAGAGATGGGTG-3′

ΔP1DelRv, 5′-CCATCTCTCTCCTTCTAGCCGGCGTACTCACCAGTCGCCG 3′.

A 2,790-bp fragment from the WT pCCL-EGFP transfer vector[47] spanning the regions between the cytomegalovirus (CMV) and hPGK (phosphoglycerate kinase) promoters was amplified using the 5′-TACGGTAAACTGCCCACTTG-3′ and 5′-TAGGTCAGGGTGGTCACGAG-3′ primers in an Accuprime *Pfx* Supermix-containing PCR reaction. The thermal cycling conditions were as follows: 95°C for 5 min; 35 cycles of 95°C for 15 s, 64°C for 30 s, and 68°C for 3 min; and a final extension step at 68°C for 7 min. The 2,790-bp PCR product was incubated with the pCRBlunt II-TOPO vector (Invitrogen) according to the manufacturer's instructions, and the resulting product was used to transform Stbl3 *E. coli*-competent cells (Invitrogen). Mutagenesis was performed using the SDM XLII Agilent kit and the primers are listed in the Materials and Methods. Mutations were verified by Sanger sequencing. The pCCL-EGFP transfer vector backbone and the mutated pCRBlunt II-TOPO vectors were digested with *Nde*I and *Mfe*I-HF, and the mutated 1081 bp *Nde*I-*Mfe*I fragment was ligated back to the digested 6,729-bp *Nde*I-*Mfe*I pCCL-EGFP backbone.

The following BglG- and MS2-containing transfer vectors were constructed:

BglGFw, 5′-[phos]-CGGGATTGTTACTGCATTCGCAGGCAAAACCCGAT-3′

BglGRv, 5′-[phos]-cGGGTTTTGCCTGCGAATGCAGTAACAATCCCGAT-3′

MS2Fw, 5′-[phos]-CGGCATGAGGATCACCCATGTCGAT-3′

MS2Rv 5′-[phos]-CGACATGGGTGATCCTCATGCCGAT-3′

Oligonucleotides were used in independent annealing reactions that contained 100 μM Fw oligo, 100 μM Rv Oligo, and 1× ExpressLink T4 buffer (Invitrogen) in a 100-μL final volume reaction that was held at 90°C for 10 min. These oligos contained the following sequences of BglG flanked between the *Pvu*I recognition sites: 5′-GGATTGTTACTGCATTCGCAGGCAAAACC-3′ and MS2: 5′-GCATGAGGATCACCCATGT-3′. Reagents were allowed to cool down to room temperature. 1 μg of each pCCL backbone of interest was digested with 20 U of FastDigest *Pvu*I (Thermo Fisher Scientific) and dephosphorylated with 1 U of FastAP thermosensitive alkaline phosphatase (Thermo Fisher Scientific) in a 20-μL reaction in 1× Fast Digest Buffer (Thermo Fisher Scientific) at 37°C for 1 h. Digestion products were separated on a 1% agarose gel, the bands of interest were excised, and DNA was extracted using a gel extraction kit (QIAGEN). Ligation reactions contained 35 ng of BglG or MS2 annealed oligos, 100 ng backbone, and 5 U of T4 expressLink ligase (Invitrogen) in a final volume of 20 μL and were incubated at room temperature for 2 h 30 min. 1 μL of each ligation reaction containing a combination of backbone and oligos was used to transform Stbl3 *E. coli* cells. Successful incorporation of the BglG or MS2 sequence in both transfer vectors was confirmed by sequencing. Circular plasmids were used for standard curve generation in qPCRs.

RNA Purification

Intracellular RNA from transfected cells was extracted using the QIAGEN RNeasy kit as described by the manufacturer. For extracellular RNA extraction, 2 mL of purified vector supernatant was centrifuged at 22,000 × *g* for 3 h at 4°C in 8.4% Opti-Prep (STEMCELL Technologies). Pellets were resuspended in phosphate-buffered saline (PBS) (Thermo Fisher Scientific) and incubated in 200 μL of proteinase K extraction buffer (50 mM Tris-HCl [pH 7.5], 100 mM NaCl, 10 mM EDTA, 1% SDS, 100 μg/mL proteinase K [Ambion], and 100 μg/mL yeast tRNA [Sigma-Aldrich]) at 37°C for 30 min. vRNA was extracted with phenol-chloroform and chloroform:isoamyl alcohol, precipitated with 1/10th volume of 3 M sodium acetate (pH 5.5) and 2.5 volumes of ethanol, washed with ice-cold 70% ethanol, and air-dried.

qRT-PCR Assays

WPRE qRT-PCR

Extracted RNA was digested with Turbo DNase (Invitrogen) according to the manufacturer's instructions and purified with phenol-

chloroform extraction and ethanol precipitation as above. The RNA concentration in each sample, which represented mainly the tRNA used for precipitation, was quantified by spectrophotometry. cDNA was synthesized using the High Capacity cDNA Reverse Transcription Kit (Applied Biosystems [AB]) and 100 ng RNA. cDNA products were quantified by qPCR using primers targeting the WPRE element (Fw, 5′-CCGTTGTCAGGCAACGTG-3′; Rv, 5′-AGCTGACAGGT GGTGGCAAT-3′; probe, 5′-FAM-TGCTGACGCAACCCCCAC TGGT-BHQ1-3′)[30] using the 7500 Fast Real-Time PCR System (Applied Biosystems). The final reaction conditions were 100 nM probe, 400 nM each primer, 1× Fast Advanced buffer (Applied Biosystems), and 10 ng cDNA in a 10-μL reaction volume. Cycling conditions were 95°C for 5 min and 40 cycles of 95°C for 15 s, 60°C for 15 s, and 72°C for 15 s. The cellular levels of β-actin cDNA were measured using the SYBR Green technology and 40 nM of the following primers: Fw, 5′-GAGCGGTTCCGCTGCCCTGAGG-CACTC-3′; Rv, 5′-GGGCAGTGATCTCCTTCTGCA TCCTG-3′.[37] The reaction conditions were 40 nM each primer, 1× SYBR Green Fast Advanced buffer (Applied Biosystems), and 10 ng cDNA in a 10-μL reaction volume. Cycling conditions were 50°C for 2 min, 95°C for 20 s, and 40 cycles of 95°C for 1 s and 60°C for 20 s. Duplicate technical repeats were included for all samples. Ct values higher than 30 were rejected.

Competitive qRT-PCR BglG/MS2 Assay

Intracellular and extracellular RNAs were isolated as described above. Removal of contaminant plasmid DNA was achieved using the Turbo DNA-free Kit (Invitrogen) in a reaction containing 8 U of Turbo DNase enzyme, 1× Turbo DNase buffer, and the total amount of RNA isolated at 37°C for 2 h. One-fifth volume of the inactivation reagent was added and incubated at room temperature for 5 min. RNA was recovered after centrifugation at 10,000 × *g* for 2 min. Reverse transcription was performed as above, and cDNAs were then quantified by qPCR using a common set of primers (Fw, 5′-GAATTCTGCA GTCGACGGTA-3′; Rv, 5′-TCCAGAGGTTGATTGCGA-3′) and probes targeting the unique sequence of BglG (5′-FAM-TCGATCGGGATTGTTACTG-BHQ1-3′) or MS2 (5′-HEX (hexachloro-fluorescein)-CATGGGT GATCCTCATGCCGAT-BHQ2-3′). Sequences and primer/probe pairs were designed using the Oligo 7.0, Molecular Biology Insights software.[48] After optimization, the final reaction conditions were 750 nM BglG probe or 250 nM MS2 probe, 500 nM each primer, 1× Fast Advanced buffer (Applied Biosystems), and 3 μL cDNA in a 10-μL reaction volume. Cycling conditions were 95°C for 5 min and 40 cycles of 95°C for 15 s, 60°C for 15 s, and 72°C for 15 s. Duplicate technical repeats were included for all samples. Ct values higher than 30 were rejected. RPEs were compared using Student's t test (2-sample, unequal variance) and power analyses (conducted in G*power 3.1[49,50] using a power of 0.95 to detect the difference between two independent means).

Northern Blotting

A 744-nucleotide PCR fragment including the EGFP open reading frame (ORF) and the start of the WPRE sequence was amplified by PCR using the following primers from pCCL-EGFP: Fw, 5′-GCTCCCTCGTTGACCGAATC-3′; Rv, 5′-TAATACGACTCA CTATAGGGTCGTCCATGCCGAGAGTGATC-3. The PCR fragment was isolated by agarose gel electrophoresis and purified (QIAGEN gel extraction kit) . *In vitro* transcription was carried out using the MEGAscript T7 transcription kit (Ambion) in a reaction containing PCR product (1 μg); 1× transcription buffer; 10 mM each ATP, CTP, and guanosine triphosphate (GTP); 6 mM UTP, 7 mM DIG (digoxigenin)-UTP, and T7 polymerase enzyme mix (2 U). RNA was treated with Turbo DNase and purified with lithium chloride precipitation.

Purified extracellular RNA samples were analyzed on ethidium bromide-stained 1% agarose gels in 0.5× Tris-borate-EDTA (TBE) (Thermo Fisher Scientific) and electrophoresed at 60 V for at least 4 h. Samples were transferred from the gel to a Hybond N+ nylon membrane (GE Healthcare) by capillary transfer for 2 h in UltraPure 20× saline sodium citrate (SSC) (Thermo Fisher Scientific).

The membrane was crosslinked by baking at 80°C for 30 min and hybridized in UltraHyb (Invitrogen), which contained 75 ng of the produced probe, overnight at 68°C. The bound probe was visualized using the DIG luminescence detection kit (Roche) following the manufacturer's protocol and the anti-DIG-AP fragment antibody (1:20,000 dilution). RNA was visualized by addition of CDP-Star and detected with photographic film.

Western Blotting

For intracellular samples, supernatant was removed 48 h after transfection, and cells were harvested and pelleted in ice-cold PBS. Cells were lysed with radioimmunoprecipitation assay (RIPA) lysis and extraction buffer (Thermo Fisher Scientific) containing 1× HALT protease inhibitor cocktail (Thermo Fisher Scientific). Protein quantification was performed using the BCA protein assay kit (Pierce) according to the manufacturer's instructions. Extracted cell lysates were heated in 1× Laemmli sample loading buffer (Sigma-Aldrich) at 95°C for 10 min. 20 μg protein per sample was analyzed by SDS-PAGE. Glyceraldehyde 3-phosphate dehydrogenase (GAPDH) used as a loading control, was detected with anti-GAPDH (1:10,000, Abcam) and donkey anti-rabbit antibody (1:2,000, Abcam).

For extracellular samples, supernatant (8 mL) was centrifuged at 3,000 × *g* for 10 min at 4°C to remove cell debris. 1 mL of the purified supernatant was subsequently centrifuged at 22,000 × *g* for 3 h at 4°C. The pellet was resuspended in PBS, and the proteins were denatured by heating to 95°C in 1× Laemmli sample loading buffer (Sigma-Aldrich). Samples (1 μL) were separated by SDS-PAGE at 100 V for 4 h. Proteins were transferred to a nitrocellulose membrane by electroblotting at 100 V for 90 min in 1× transfer buffer (25 mM Tris, 192 mM glycine, 20% methanol [v/v], and 0.1% SDS) before blocking in 5% (w/v) skimmed milk powder in PBS for 30 min. The primary antibody for p24, ARP313 (NIBSC), was incubated overnight at 4°C at 1:20,000 dilution in block, followed by three 10-min washes with PBST (0.1% Tween in PBS). A secondary antibody (horse anti-mouse horseradish peroxidase [HRP], Abcam) was then used

at 1:2,000 for 2 h. The membrane was washed as described previously and developed with ECL Prime (GE Healthcare) and exposure to photographic film. The films were scanned, and bands were quantified using ImageJ.[51]

ELLA p24 Assay

1 mL of supernatant was harvested 40 h after transfection, centrifuged at 10,000 × g for 10 min at 4°C to remove any residual debris, and stored at −80°C. Samples were thawed at room temperature for 30 min, and 500× dilutions were performed using 0.5% Triton X-100 lysis buffer (ProteinSimple). 1 mL of the wash buffer (ProteinSimple) was loaded in each of the designated wells, and 50 μL of the diluted samples were loaded on the ELLA cartridge (ProteinSimple). The run lasted 90 min, and the software automatically generated the final p24 results in pg/mL based on the raw data and the built-in standard curve.

SUPPLEMENTAL INFORMATION

Supplemental Information can be found online at https://doi.org/10.1016/j.omtm.2020.09.010.

AUTHOR CONTRIBUTIONS

C.A.V. and A.M.L.L. conceived the project. E.V., C.A.V., A.M.L.L., and J.C.K. designed the study. E.V. performed the experiments and analyzed the data under the supervision of J.C.K., C.A.V., and A.M.L.L. E.V. generated the original draft manuscript and designed the figures with guidance from J.C.K. E.V., C.A.V., A.M.L.L., and J.C.K. reviewed and edited the manuscript. All authors read and approved the final version.

CONFLICTS OF INTEREST

The authors declare no competing interests.

ACKNOWLEDGMENTS

We are thankful to Dr. Triantafilos Liloglou for help with designing the primer and probe sequences. We would also like to thank Dr. Stephen Graham and Dr. Nick Matheson for their original ideas regarding the competitive qPCR assay and its design. Last, we thank Dr. Harriet Groom and Bhupal Auothu for technical expertise and help with qPCR and ELLA, respectively. E.V. was supported by a grant from the BBSRC (BB/N503708/1 to A.M.L.L. and C.A.V.). A.M.L.L. is supported by the Clinical Academic Reserve and his laboratory by the Cambridge Biomedical Research Centre.

REFERENCES

1. Coffin, J.M., Hughes, S.H., and Varmus, H. (1997). Retroviruses (Cold Spring Harbor Laboratory Press).
2. Los Alamos National Laboratory. HIV Sequence Compendium 2016. https://www.hiv.lanl.gov/content/sequence/HIV/COMPENDIUM/2016compendium.html.
3. Krebs, F.C., Hogan, T.C., Quiterio, S., Gartner, S., and Wigdahl, B. Lentiviral LTR-directed Expression, Sequence Variation, and Disease Pathogenesis. https://www.hiv.lanl.gov/content/sequence/HIV/REVIEWS/WIGDAHL2001/Wigdahl.html.
4. Baudin, F., Marquet, R., Isel, C., Darlix, J.L., Ehresmann, B., and Ehresmann, C. (1993). Functional sites in the 5′ region of human immunodeficiency virus type 1 RNA form defined structural domains. J. Mol. Biol. *229*, 382–397.
5. Kuzembayeva, M., Dilley, K., Sardo, L., and Hu, W.S. (2014). Life of psi: how full-length HIV-1 RNAs become packaged genomes in the viral particles. Virology *454-455*, 362–370.
6. Laughrea, M., Jetté, L., Mak, J., Kleiman, L., Liang, C., and Wainberg, M.A. (1997). Mutations in the kissing-loop hairpin of human immunodeficiency virus type 1 reduce viral infectivity as well as genomic RNA packaging and dimerization. J. Virol. *71*, 3397–3406.
7. Berkhout, B., and van Wamel, J.L. (1996). Role of the DIS hairpin in replication of human immunodeficiency virus type 1. J. Virol. *70*, 6723–6732.
8. Feng, Y.X., Copeland, T.D., Henderson, L.E., Gorelick, R.J., Bosche, W.J., Levin, J.G., and Rein, A. (1996). HIV-1 nucleocapsid protein induces and quot;maturationand quot; of dimeric retroviral RNA in vitro. Proc. Natl. Acad. Sci. USA *93*, 7577–7581.
9. Clever, J., Sassetti, C., and Parslow, T.G. (1995). RNA secondary structure and binding sites for gag gene products in the 5′ packaging signal of human immunodeficiency virus type 1. J. Virol. *69*, 2101–2109.
10. Sakuragi, J., Iwamoto, A., and Shioda, T. (2002). Dissociation of genome dimerization from packaging functions and virion maturation of human immunodeficiency virus type 1. J. Virol. *76*, 959–967.
11. Russell, R.S., Hu, J., Bériault, V., Mouland, A.J., Laughrea, M., Kleiman, L., Wainberg, M.A., and Liang, C. (2003). Sequences downstream of the 5′ splice donor site are required for both packaging and dimerization of human immunodeficiency virus type 1 RNA. J. Virol. *77*, 84–96.
12. Olson, E.D., Cantara, W.A., and Musier-Forsyth, K. (2015). New structure sheds light on selective HIV-1 genomic RNA packaging. Viruses *7*, 4826–4835.
13. Ooms, M., Huthoff, H., Russell, R., Liang, C., and Berkhout, B. (2004). A riboswitch regulates RNA dimerization and packaging in human immunodeficiency virus type 1 virions. J. Virol. *78*, 10814–10819.
14. Lu, K., Heng, X., and Summers, M.F. (2011). Structural determinants and mechanism of HIV-1 genome packaging. J. Mol. Biol. *410*, 609–633.
15. Abbink, T.E.M., and Berkhout, B. (2003). A novel long distance base-pairing interaction in human immunodeficiency virus type 1 RNA occludes the Gag start codon. J. Biol. Chem. *278*, 11601–11611.
16. Lu, K., Heng, X., Garyu, L., Monti, S., Garcia, E.L., Kharytonchyk, S., Dorjsuren, B., Kulandaivel, G., Jones, S., Hiremath, A., et al. (2011). NMR detection of structures in the HIV-1 5′-leader RNA that regulate genome packaging. Science *334*, 242–245.
17. Escors, D., and Breckpot, K. (2010). Lentiviral vectors in gene therapy: their current status and future potential. Arch. Immunol. Ther. Exp. (Warsz.) *58*, 107–119.
18. Aiuti, A., Biasco, L., Scaramuzza, S., Ferrua, F., Cicalese, M.P., Baricordi, C., Dionisio, F., Calabria, A., Giannelli, S., Castiello, M.C., et al. (2013). Lentiviral Hematopoietic Stem Cell Gene Therapy in Patients with Wiskott-Aldrich Syndrome. Science *341*, 1233151.
19. Biffi, A., Montini, E., Lorioli, L., Cesani, M., Fumagalli, F., Plati, T., Baldoli, C., Martino, S., Calabria, A., Canale, S., et al. (2013). Lentiviral Hematopoietic Stem Cell Gene Therapy Benefits Metachromatic Leukodystrophy. Science *341*, 1233158.
20. Aiuti, A., Biasco, L., Scaramuzza, S., Ferrua, F., Cicalese, M.P., Baricordi, C., Dionisio, F., Calabria, A., Giannelli, S., Castiello, M.C., et al. (2013). Lentiviral Hematopoietic Stem Cell Gene Therapy in Patients with Wiskott-Aldrich Syndrome. Science *341*, 1233151.
21. Segura, M.M., Mangion, M., Gaillet, B., and Garnier, A. (2013). New developments in lentiviral vector design, production and purification. Expert Opin. Biol. Ther. *13*, 987–1011.
22. Dorman, N., and Lever, A. (2000). Comparison of viral genomic RNA sorting mechanisms in human immunodeficiency virus type 1 (HIV-1), HIV-2, and Moloney murine leukemia virus. J. Virol. *74*, 11413–11417.
23. Butsch, M., and Boris-Lawrie, K. (2000). Translation is not required To generate virion precursor RNA in human immunodeficiency virus type 1-infected T cells. J. Virol. *74*, 11531–11537.
24. Kenyon, J.C., Prestwood, L.J., Le Grice, S.F., and Lever, A.M.L. (2013). In-gel probing of individual RNA conformers within a mixed population reveals a dimerization structural switch in the HIV-1 leader. Nucleic Acids Res. *41*, e174.
25. Ferrer, M., Henriet, S., Chamontin, C., Lainé, S., and Mougel, M. (2016). From Cells to Virus Particles: Quantitative Methods to Monitor RNA Packaging. Viruses *8*, 239.

26. Sakuragi, J., Shioda, T., and Panganiban, A.T. (2001). Duplication of the primary encapsidation and dimer linkage region of human immunodeficiency virus type 1 RNA results in the appearance of monomeric RNA in virions. J. Virol. *75*, 2557–2565.

27. Nikolaitchik, O.A., Dilley, K.A., Fu, W., Gorelick, R.J., Tai, S.H., Soheilian, F., Ptak, R.G., Nagashima, K., Pathak, V.K., and Hu, W.S. (2013). Dimeric RNA recognition regulates HIV-1 genome packaging. PLoS Pathog. *9*, e1003249.

28. (2013). The Mfold Web Server. http://unafold.rna.albany.edu/?q=mfold.

29. Los Alamos National Laboratory. HIV-1 Gene Map. https://www.hiv.lanl.gov/content/sequence/HIV/MAP/landmark.html.

30. Lizée, G., Aerts, J.L., Gonzales, M.I., Chinnasamy, N., Morgan, R.A., and Topalian, S.L. (2003). Real-time quantitative reverse transcriptase-polymerase chain reaction as a method for determining lentiviral vector titers and measuring transgene expression. Hum. Gene Ther. *14*, 497–507.

31. Zufferey, R., Donello, J.E., Trono, D., and Hope, T.J. (1999). Woodchuck hepatitis virus posttranscriptional regulatory element enhances expression of transgenes delivered by retroviral vectors. J. Virol. *73*, 2886–2892.

32. Scherr, M., Battmer, K., Blömer, U., Ganser, A., and Grez, M. (2001). Quantitative determination of lentiviral vector particle numbers by real-time PCR. Biotechniques *31*, 520–526, 522, 524 passim.

33. Sastry, L., Johnson, T., Hobson, M.J., Smucker, B., and Cornetta, K. (2002). Titering lentiviral vectors: comparison of DNA, RNA and marker expression methods. Gene Ther. *9*, 1155–1162.

34. Butler, S.L., Hansen, M.S.T., and Bushman, F.D. (2001). A quantitative assay for HIV DNA integration in vivo. Nat. Med. *7*, 631–634.

35. Charrier, S., Stockholm, D., Seye, K., Opolon, P., Taveau, M., Gross, D.A., Bucher-Laurent, S., Delenda, C., Vainchenker, W., Danos, O., and Galy, A. (2005). A lentiviral vector encoding the human Wiskott-Aldrich syndrome protein corrects immune and cytoskeletal defects in WASP knockout mice. Gene Ther. *12*, 597–606.

36. Van Maele, B., De Rijck, J., De Clercq, E., and Debyser, Z. (2003). Impact of the central polypurine tract on the kinetics of human immunodeficiency virus type 1 vector transduction. J. Virol. *77*, 4685–4694.

37. Duffy, S., and Cochrane, A. (2012). Analysis of HIV-1 RNA Splicing. In Alternative pre-mRNA Splicing, S. Stamm, C.J. Smith, and R. Luhrmann, eds. (Wiley), pp. 438–448.

38. Bell, N.M., and Lever, A.M.L. (2013). HIV Gag polyprotein: processing and early viral particle assembly. Trends Microbiol. *21*, 136–144.

39. Moore, M.D., Nikolaitchik, O.A., Chen, J., Hammarskjöld, M.L., Rekosh, D., and Hu, W.S. (2009). Probing the HIV-1 genomic RNA trafficking pathway and dimerization by genetic recombination and single virion analyses. PLoS Pathog. *5*, e1000627.

40. Bustin, S.A., Benes, V., Garson, J.A., Hellemans, J., Huggett, J., Kubista, M., Mueller, R., Nolan, T., Pfaffl, M.W., Shipley, G.L., et al. (2009). The MIQE guidelines: minimum information for publication of quantitative real-time PCR experiments. Clin. Chem. *55*, 611–622.

41. Applied Biosystems. TaqMan MGB Probe. https://www.thermofisher.com/order/catalog/product/4316034#/4316034.

42. Spiess, A.-N., and Ivell, R. (2002). A highly efficient method for long-chain cDNA synthesis using trehalose and betaine. Anal. Biochem. *301*, 168–174.

43. Lever, A., Gottlinger, H., Haseltine, W., and Sodroski, J. (1989). Identification of a sequence required for efficient packaging of human immunodeficiency virus type 1 RNA into virions. J. Virol. *63*, 4085–4087.

44. Ingemarsdotter, C.K., Zeng, J., Long, Z., Lever, A.M.L., and Kenyon, J.C. (2018). An RNA-binding compound that stabilizes the HIV-1 gRNA packaging signal structure and specifically blocks HIV-1 RNA encapsidation. Retrovirology *15*, 25.

45. van Bel, N., Ghabri, A., Das, A.T., and Berkhout, B. (2015). The HIV-1 leader RNA is exquisitely sensitive to structural changes. Virology *483*, 236–252.

46. Agilent. QuikChange Primer Design. https://www.agilent.com/store/primerDesignProgram.jsp.

47. Dull, T., Zufferey, R., Kelly, M., Mandel, R.J., Nguyen, M., Trono, D., and Naldini, L. (1998). A third-generation lentivirus vector with a conditional packaging system. J. Virol. *72*, 8463–8471.

48. Rychlik, W. (2007). OLIGO 7 primer analysis software. Methods Mol. Biol. *402*, 35–60.

49. Faul, F., Erdfelder, E., Lang, A.G., and Buchner, A.G. (2007). Power 3: A flexible statistical power analysis program for the social, behavioral, and biomedical sciences. Behav. Res. Methods *39*, 175–191.

50. Sinck, L., Richer, D., Howard, J., Alexander, M., Purcell, D.F., Marquet, R., and Paillart, J.C. (2007). In vitro dimerization of human immunodeficiency virus type 1 (HIV-1) spliced RNAs. RNA *13*, 2141–2150.

51. National Institutes of Health. ImageJ. https://imagej.nih.gov/ij/index.html.

OMTM, Volume *19*

Supplemental Information

Development of a Novel Competitive qRT-PCR Assay to Measure Relative Lentiviral Packaging Efficiency

Eirini Vamva, Andrew M.L. Lever, Conrad A. Vink, and Julia C. Kenyon

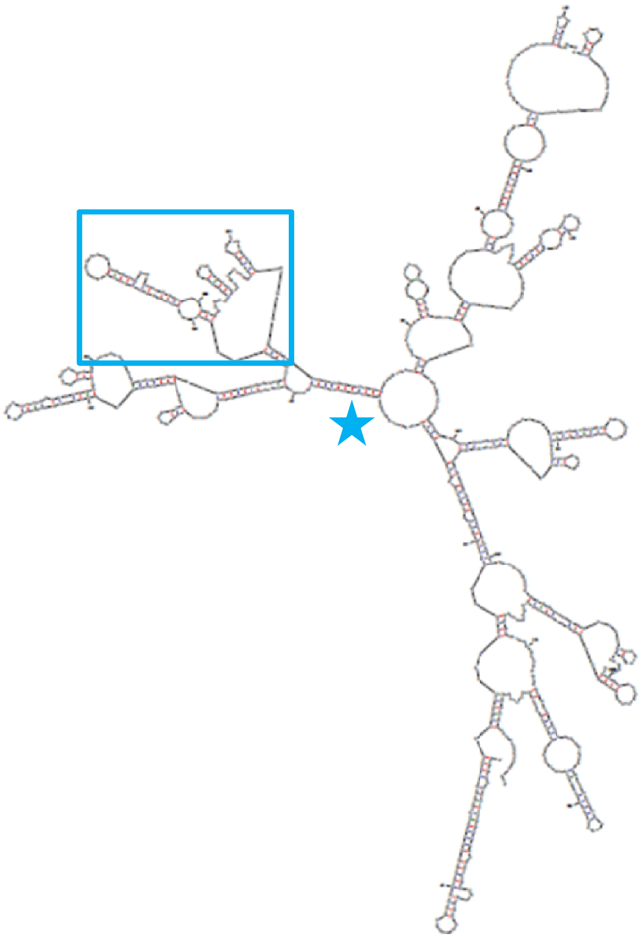

U5s (ΔG=-240.6 kJ/mol)

mFold predicted secondary structures of U5s mutant

Sup1

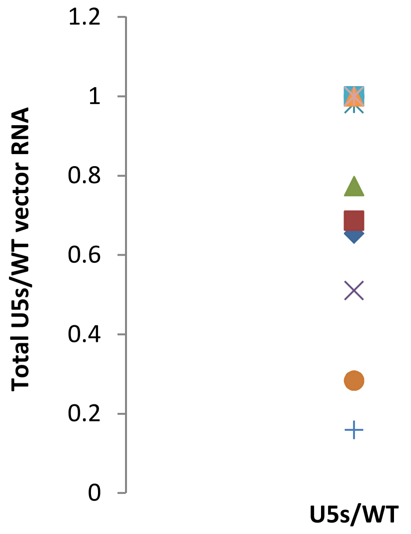

Alternative way of presenting U5s/WT packaging efficiency data from Northern blots in the form of individual data points.

Sup2

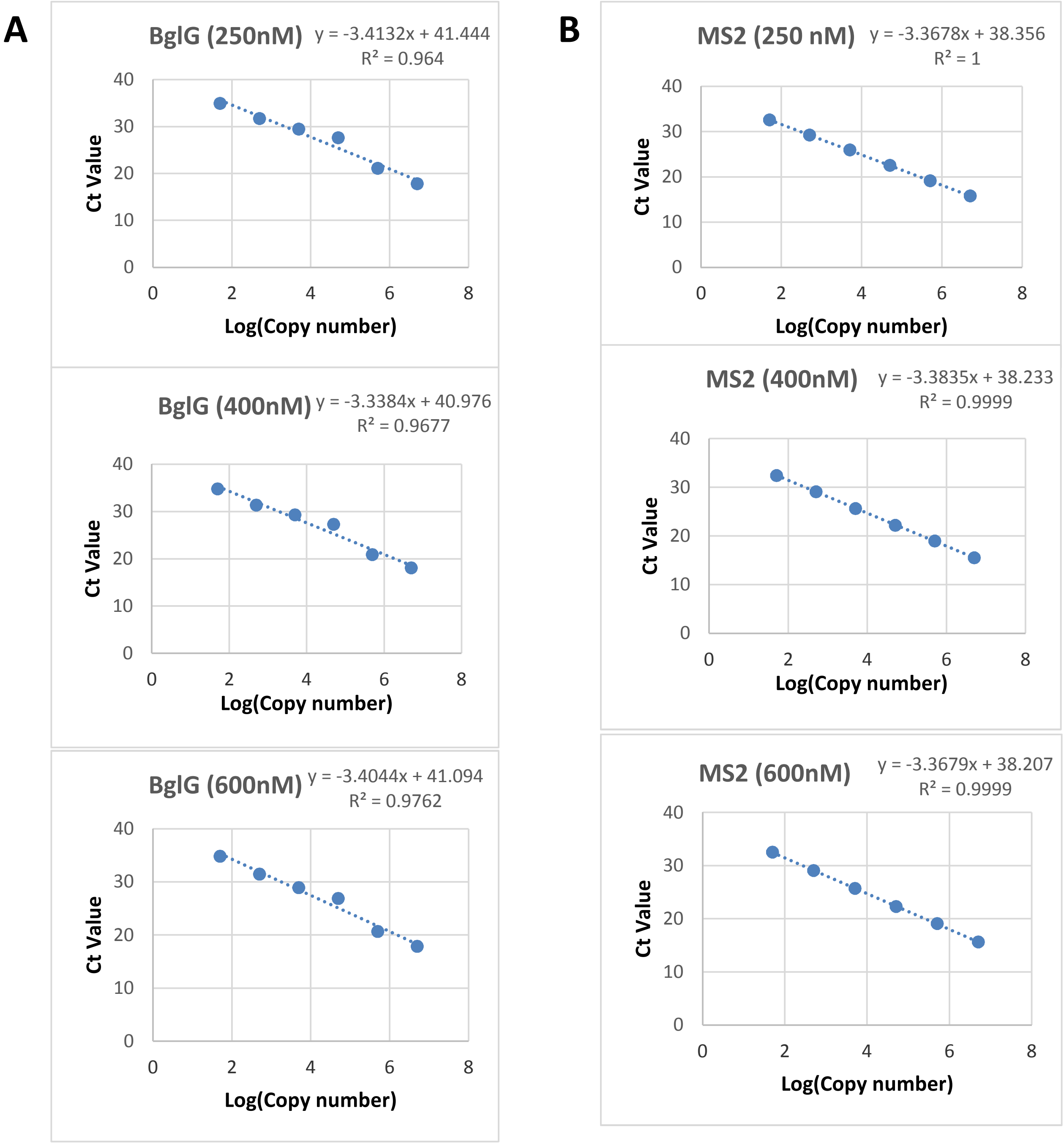

Optimization of [A] BglG and [B] MS2 probes' concentration

Sup3

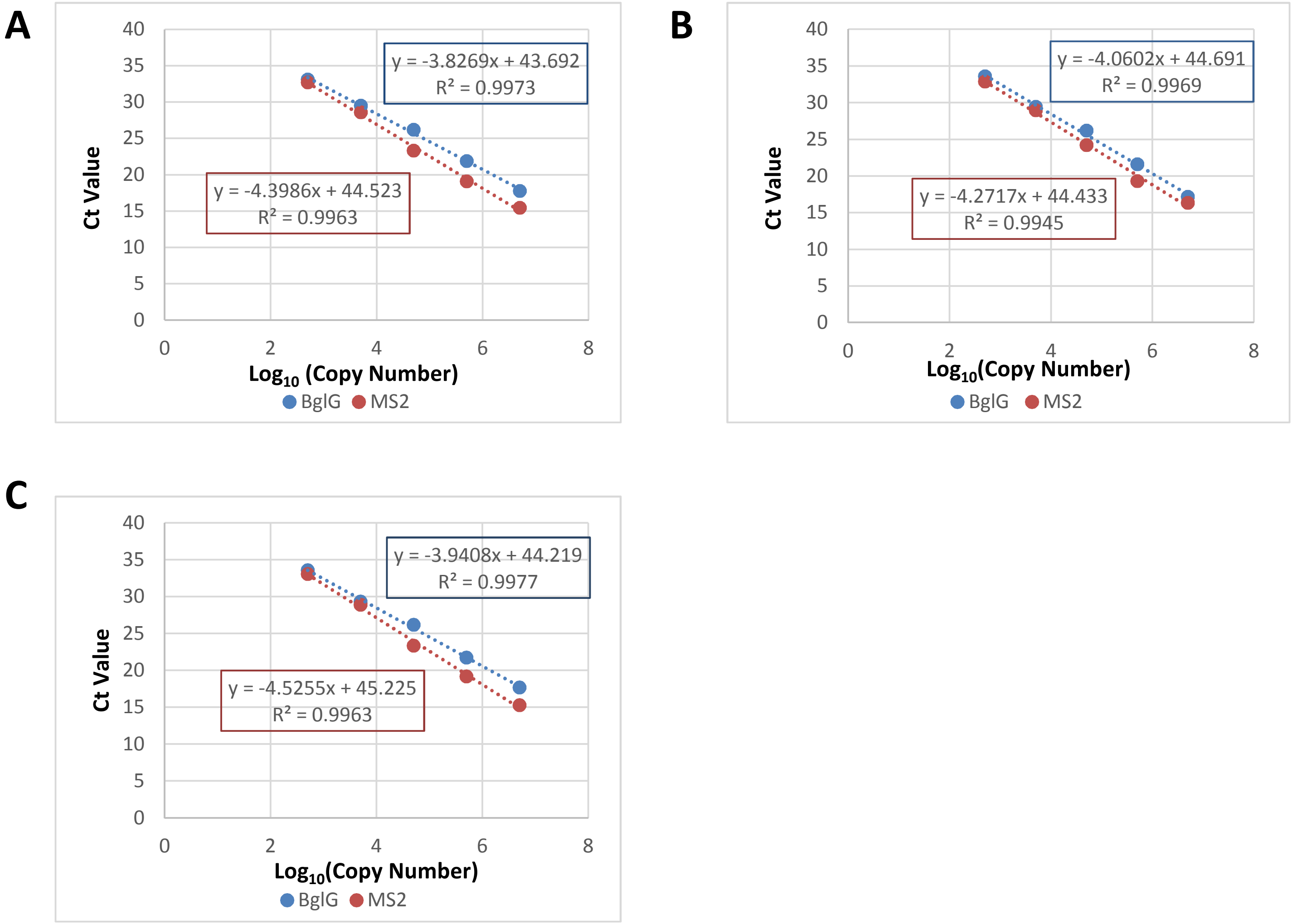

Multiplex reaction set up with 750 nM new AB BglG probe, 250 nM MS2 probe, 500 nM Forward primer, 500 nM Reverse primer and 5 µl Fast Advanced Buffer. [B] Multiplex reaction set up with 750 nM new AB BglG probe, 250 nM MS2 probe, 1000 nM Forward primer, 1000 nM Reverse primer and 5 µl Fast Advanced Buffer. [C] Multiplex reaction set up with 750 nM new AB BglG probe, 250 nM MS2 probe, 1500 nM Forward primer, 1500 nM Reverse primer and 5 µl Fast Advanced Buffer. Experiments shown are representative of 3 independent replicates, each using two technical repeats. The linearity of the 1000 nM MS2 assay (red) increased with increasing primer concentration whereas its efficiency decreased. On the contrary, the efficiency of the 1000nM BglG assay (blue) increased with increasing primer concentration, while the linearity of the standard curve decreased. The increase of primer concentration to 1500 nM positively affected linearity but negatively affected efficiency in both assays.

Sup4

Advantage / Technique	Length and integrity of target RNA	Sensitivity and accuracy	Throughput	Measurement of multiple RNA species	Level of internal control of the assay
Northern blot	+				
Ribonuclease protection assay (RPA)		+	+	+	
Standard RT-qPCR assay		++	++		
Competitive RT-qPCR assay		++	++	++	++

Comparison between techniques commonly used for measurement of RNA packaging efficiency and our new competitive RT-qPCR assay

Sup5

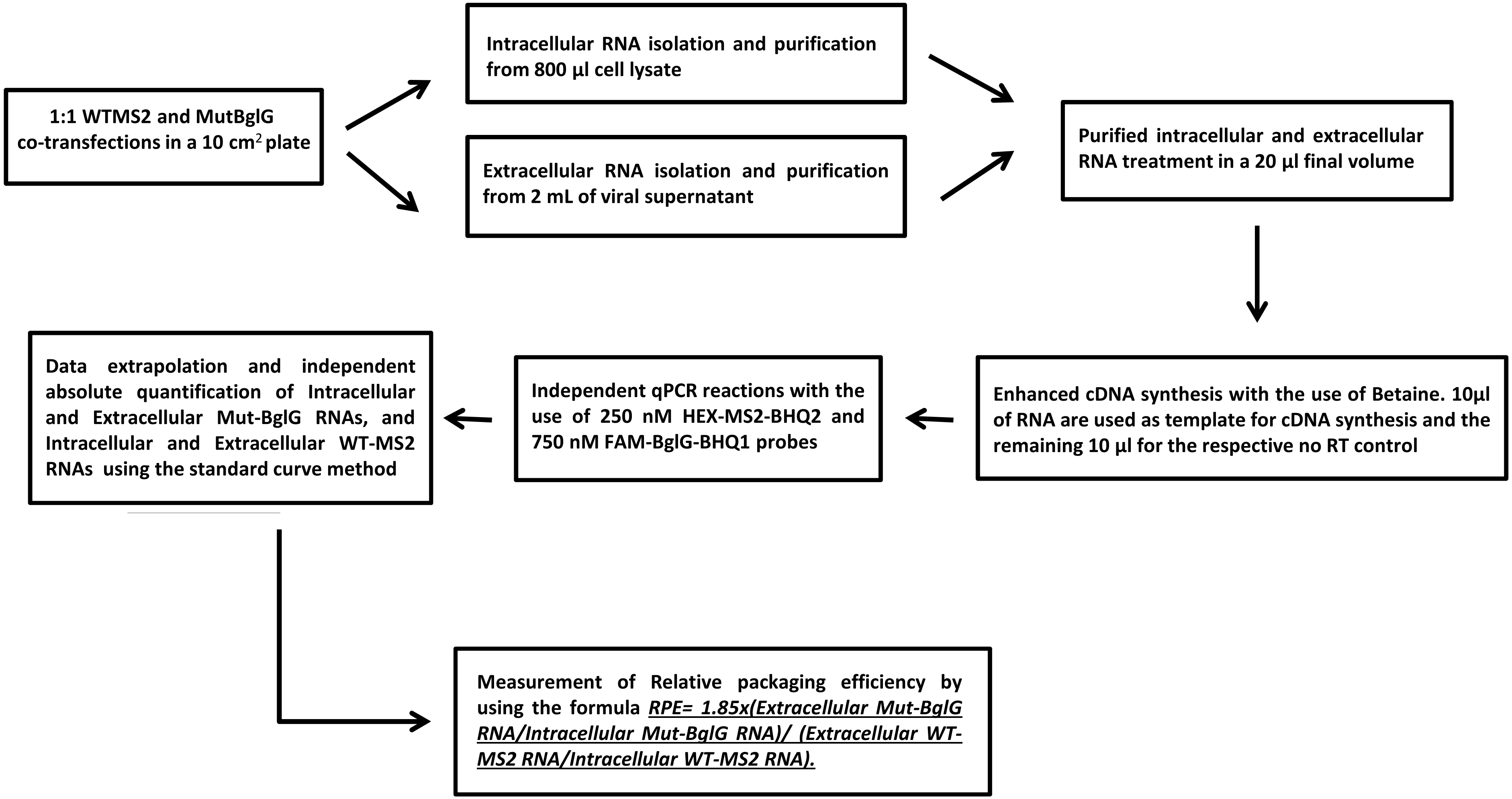

Flowchart of process required to measure RPE accurately

Sup6
